# Ascites reprograms innate lymphoid immune cells in ovarian cancer by promoting ILC2 enrichment and dysfunctional NK-cell states

**DOI:** 10.1136/jitc-2026-015396

**Published:** 2026-08-26

**Authors:** Bilal Alashkar Alhamwe, Fahd Alhamdan, Viviane Ponath, Nathalie Hoffmann, Celina Meena, Florian Fingernagel, Holger Garn, Kim Pauk, Corinna Keber, Carsten Denkert, Silke Reinartz, Elke Pogge von Strandmann

**Affiliations:** 1 Marburg University, Institute for Tumor Immunology, Marburg, Germany; 2 Department of Anesthesiology, Critical Care, and Pain Medicine, Cardiac Anesthesia Division, Boston Children’s Hospital, Department of Immunology and Anaesthesia, Harvard Medical School, Boston, Massachusetts, USA; 3 Core facility Genomics, Marburg University, Marburg, Germany; 4 Marburg University, Translational Inflammation Research Division & Core Facility for Single Cell Multiomics, Medical Faculty, Marburg, Germany; 5 Marburg University and University Hospital Marburg (UKGM), Institute of Pathology, Marburg, Germany; 6 Marburg University, Translational Oncology, Center for Tumor Biology and Immunology (ZTI), Marburg, Germany; 7 Marburg University, Core Facility Extracellular Vesicles EV-iTEC, Marburg, Germany

**Keywords:** Ovarian Cancer

## Abstract

**Background:**

High-grade serous ovarian cancer (HGSOC) is commonly accompanied by malignant ascites, a clinically relevant tumor niche that promotes immune evasion, metastasis, and treatment resistance. Although natural killer (NK)-cell dysfunction has been described in ovarian cancer, the broader innate lymphoid landscape of ascites and the mechanisms linking ascites-derived signals to innate immune suppression remain insufficiently resolved.

**Methods:**

We performed single-cell RNA sequencing of NK/innate lymphoid cells from ovarian cancer ascites to define cellular heterogeneity and differentiation states. Functional assays assessed NK-cell cytotoxicity, degranulation, and receptor expression following exposure to patient-derived ascites, with or without transforming growth factor-β (TGF-β) receptor inhibition. Proteomic profiling was used to characterize the soluble ascites milieu, and clinical associations were examined for innate lymphoid subsets.

**Results:**

Single-cell analysis identified eight transcriptionally distinct NK/innate lymphoid states, including cytotoxic, precursor, early-like, tolerant/immunoregulatory, regulatory, proinflammatory, and innate lymphoid populations. Ovarian cancer ascites was characterized by depletion of cytotoxic and precursor NK-cell states together with enrichment of early-like, tolerant, regulatory, pro-inflammatory, and innate lymphoid cell (ILC) populations. Trajectory analysis indicated impaired maturation toward terminally differentiated cytotoxic NK cells. Notably, ascites contained an expanded population of programmed cell death protein 1 (PD-1)^+^ ILC2s, which were more abundant in patients with shorter progression-free survival. In functional assays, short-term exposure of healthy donor NK cells to ascites suppressed degranulation and tumor-cell killing, reduced expression of activating receptors including NKp30 and DNAM-1, increased inhibitory receptor expression, and shifted NK cells toward a CD56^high^CD16^low^ phenotype. Proteomic profiling supported a soluble milieu consistent with type 2 immune skewing and NK-cell suppression. Importantly, TGF-β receptor inhibition partially restored NK-cell activation and function in the presence of ascites.

**Conclusions:**

HGSOC ascites establishes a type 2-skewed immunoregulatory niche that coordinately drives NK cell dysfunction and PD-1^+^ ILC2 accumulation. The findings identify TGF-β-linked suppression and ascites-associated immune regulators as candidate immunotherapeutic vulnerabilities for restoring antitumor immunity in ovarian cancer.

**WHAT IS ALREADY KNOWN ON THIS TOPIC**

- Malignant ascites constitutes an immunosuppressive microenvironment in ovarian cancer and impairs natural killer (NK)-cell function. However, the heterogeneity of ascites-associated NK cells and innate lymphoid cells (ILCs), the mechanisms underlying their dysfunction, and their clinical relevance remain incompletely understood.

**WHAT THIS STUDY ADDS**

- The combination of single-cell RNA sequencing, flow cytometry, functional NK assays, and proteomics demonstrates that ovarian cancer ascites promotes dysfunctional NK-cell states and the accumulation of programmed cell death protein 1 (PD-1)-positive group 2 ILC2s. Transforming growth factor-β contributes to ascites-induced NK-cell dysfunction, while increased ILC2 abundance and PD-1 expression are associated with shorter progression-free survival.

**HOW THIS STUDY MIGHT AFFECT RESEARCH, PRACTICE OR POLICY**

- By providing a comprehensive characterization of NK-cell and ILC2 states in ovarian cancer ascites and identifying an association between PD-1-positive ILC2 enrichment and shorter progression-free survival, this study provides a rationale for the development of therapeutic strategies targeting ascites-driven innate immune dysfunction.

## Background

 High-grade serous ovarian cancer (HGSOC) is the leading cause of mortality among women diagnosed with gynecologic malignancies, accounting for approximately 70% of epithelial OCs and the majority of OC-related deaths.^1–3^ Late diagnosis, tumor heterogeneity, and aggressive metastasis contribute to the low 5-year survival rate of 28–40%, with 70% of patients presenting with peritoneal metastases and malignant ascites at diagnosis.^4–7^ Chemotherapy combined with surgical removal is considered the standard treatment for HGSOC; however, recurrence rates are high, and responses to treatment remain poor.^8 9^ The peritoneal dissemination of tumor cells and the accumulation of malignant ascites are hallmarks of advanced HGSOC, generating a tumor-permissive environment that limits the efficacy of treatment.^10 11^ This underscores the urgent need for novel therapeutic approaches capable of overcoming the immunosuppressive tumor microenvironment (TME) and improving patient outcomes. Understanding the impact of ascites on innate immune cells, such as natural killer (NK) cells and innate lymphoid cells (ILCs), is essential for patient stratification, identifying immune states linked to therapeutic vulnerability, and developing strategies to reverse the immunosuppressive phenotype driven by the ascitic TME. In this context, defining how ascites shapes innate immune cells, such as NK cells and ILCs, is essential for understanding disease progression and identifying candidate targets and immune features associated with ascites-driven immune suppression.

ILCs, including NK cells, are an important arm of the innate immune system against cancer progression.^12 13^ NK cells account for approximately 5–20% of peripheral blood lymphocytes^12 14^ and play essential roles in tumor regression and the elimination of malignant cells.^12 15 16^ Functional impairment of NK cells correlates with increased cancer incidence and metastatic dissemination.^17^ NK cells exert antitumor activity via perforin-mediated and granzyme-mediated cytotoxicity and through the production of proinflammatory cytokines such as interferon-gamma (*IFNG*) and tumor necrosis factor (*TNF*).^18–20^ Based on cluster of differentiation CD56 and CD16 expression, human NK cells are classified into two principal subsets. CD56^high^CD16^low^ NK cells, comprising ~10–15% of circulating NKs, have limited direct cytotoxic capacity but produce abundant cytokines, including IFNG and TNF, thereby contributing predominantly to immune regulation within the TME. In contrast, CD56^low^CD16^high^ NK cells, which constitute ~90% of circulating NKs, are highly cytotoxic, mediating perforin-dependent and granzyme-dependent tumor killing and antibody-dependent cellular cytotoxicity (ADCC).^21–25^ Furthermore, transcriptional profiling distinguishes NK cells into cytotoxic and tolerance-associated subsets, reflecting their functional adaptation to the TME.^24^ However, the phenotypic diversity and functional states of NK cells in HGSOC ascites, and how these subsets are reprogrammed by the ascitic TME, remain incompletely defined.

ILCs represent a heterogeneous arm of the innate immune system with key roles in responses to allergens and pathogens.^26^ In cancer, ILCs can exert either tumor-promoting or tumor-suppressive effects, largely dictated by the TME.^27^ They are classified into three main subsets (ILC1s, ILC2s, and ILC3s) distinguished by their transcription factor expression dependencies and cytokine profiles, mirroring T helper 1 (Th1), 2 (Th2), and 17 (Th17) cells, respectively.^27^ ILC1s, dependent on T-box transcription factor, produce TNF and IFNgamma and share functional and transcriptional similarities with NK cells.^28^ Depending on the context, ILC1s can mediate either antitumor or protumor activities, underscoring the influence of the TME on their function.^29^ ILC2s, driven by GATA-binding protein 3 (GATA3), secrete interleukin (IL-) 4, IL-5, and IL-13, and have been implicated in promoting cancer progression, invasion, and metastasis across multiple tumor types.^30–33^ Notably, tumors enriched in programmed cell death protein 1 (PD-1) positive ILC2s exhibit accelerated growth, whereas PD-1 blockade reduces tumor burden.^34^ ILC3s, regulated by retinoic acid-related orphan receptor gamma t, produce IL-17 and IL-22 and contribute to mucosal immunity.^35^ Despite growing evidence of their involvement in cancer, the phenotypic characteristics, relative abundance, functional roles, and prognostic significance of ascitic ILC1s and ILC2s in HGSOC remain largely undefined, limiting their integration into emerging immunological frameworks and their potential relevance for therapeutic targeting.

In ovarian cancer (OC), malignant ascites facilitates metastasis development by generating a unique immunosuppressive TME composed of tumor cells, stromal elements, and an acellular compartment.^36^ This particular milieu supports the dissemination and peritoneal seeding of OC cells.^37^ Beyond its structural role, ascitic fluid actively reprograms both immune and non-immune cells through tumor-derived cytokines and metabolites.^36 38–41^ Such reprogramming drives phenotypic reshaping and functional impairment of NK cells, including reduced NKp30 or DNAM-1 mediated activation and increased expression of exhaustion markers.^42–44^ These alterations may also influence the proportions and phenotypic states of ascitic ILC1s and ILC2s, but the precise impact of HGSOC ascites on these innate lymphocyte subsets, and their relationship to clinical outcome, is poorly understood.

Our study employed single-cell RNA sequencing (scRNA**-**seq) to comprehensively profile NK cells and ILCs from HGSOC ascites and healthy donor (HD) peripheral blood. Flow cytometry validated extensive ascites-driven remodeling of the innate lymphocyte compartment, marked by altered NK cell subset distribution, reduced cytotoxicity and maturation of CD56^low^CD16^high^ NK cells, and phenotypic features associated with exhaustion markers within the ascitic TME. Furthermore, we identified an increased frequency of ILC2s with higher PD-1 expression levels in ascites from patients with HGSOC with shorter survival. Collectively, this work defines the phenotypic and functional landscape of NK/ILCs in the ascitic TME, revealing potential immune features and candidate immunotherapeutic targets to restore antitumor immunity in HGSOC.

## Methods

### Single-cell sequencing and library preparation of NK cells derived from OC ascites and HD blood

Peripheral blood mononuclear cells (PBMCs) were isolated from buffy coats of HDs (n=3) using density gradient centrifugation. Mononuclear peritoneal cells (PCs) were isolated from ascitic fluid collected from patients with OC (n=6). NK cells were subsequently purified from both PBMCs and PCs using positive and negative selection NK cell isolation kit from Miltenyi and according to the manufacturer’s protocol (Miltenyi Biotec, Bergisch Gladbach, Germany). The isolated NK cells were then subjected to scRNA-seq using the BD Rhapsody Single-Cell Analysis System (Becton Dickinson, San Diego, California, USA), following the manufacturer’s protocol (Doc ID: 210966).

Single-cell multiomics was performed using the BD Rhapsody system according to the manufacturer’s protocols. All reagents and equipment were obtained from Becton Dickinson (San Diego, California, USA) if not otherwise stated. The provided cells were processed according to the Rhapsody System Single-Cell Capture and cDNA Synthesis with BD Rhapsody Single-Cell Analysis System Protocol (Doc ID: 23–22951(02)) until complementary DNA (cDNA) generation. Cells were stained with 2 mM Calcein AM (Cat. No. C1430; Thermo Fisher Scientific, Dreieich, Germany) and 0.3 mM Draq7 (Cat. No. 564904) for viability evaluation. Briefly, each single cell suspension was incubated with an individual oligo-labeled multiplex tag antibody provided with the BD Human Single Cell Sample Multiplexing Kit (Cat. No. 633781). Subsequently, labeled cell suspensions were pooled. This cell pool was then incubated with oligo-labeled AbSeq antibodies directed to six surface markers (see online supplemental table 1). About 42,000 cells were loaded onto the BD Rhapsody Cartridge (Cat. No. 400000847) followed by loading with Enhanced Cell Capture Beads (Cat. No. 664887). After cell lysis, the beads with attached cell-specific messenger RNAs (mRNAs) were extracted, and cDNA was generated. Of all captured cells, 28,000 cells were subsampled for library preparation. Libraries were prepared according to the BD Rhapsody System mRNA whole transcriptome analysis (WTA) BD AbSeq, and Sample Tag Library Preparation Protocol (Doc ID: 23–24120(02)). All procedures were performed according to protocol instructions, with eight cycles for Abseq and WTA Index PCR, according to cell numbers in the experiment. Multiplex Tag libraries were generated with 10 cycles for PCR1, according to cell numbers and 7 cycles for Index PCR, according to library concentration.

Finished libraries were sequenced on a NovaSeq 6000 Sequencer on an S1 Flowcell with 1% PhiX, allocating 44,000 reads/cell for the WTA, 1,560 reads/cell for the Abseq and 300 reads/cells for the Multiplex Tags. All antibodies used were reported in online supplemental table 1.

### Bioinformatic analysis

Raw sequencing data were processed using the BD Rhapsody Targeted Analysis Pipeline (Revision 15) on the Seven Bridges platform to generate gene expression matrices. Downstream single-cell analyses were performed in R using Seurat (V.5.2.1).

No fixed mitochondrial transcript threshold (eg, 10%) was applied; instead, cells with fewer than 200 detected genes were excluded, and genes detected in fewer than 3 cells were removed. Of 28,000 isolated cells, 15,981 passed filtering and were retained for analysis. Among these, 7,727 cells were annotated as NK cells based on canonical NK cell marker gene expression, including granulysin (GNLY) and Neural Cell Adhesion Molecule 1 (NCAM1).

For preprocessing, filtered data were normalized using Seurat’s NormalizeData function with the “LogNormalize” method and a scale factor of 10,000, resulting in log-transformed normalized expression values (log1 p-transformed counts per 10,000 reads), followed by identifying highly variable genes using FindVariableFeatures with the “vst” selection method, selecting the top 2,000 variable genes for downstream dimensionality reduction. Data were scaled using Scaledata (Seurat V.5.2.1) on variable features using default parameters. After metadata harmonization, individual Seurat objects were merged for downstream analysis.

Dimensionality reduction, graph-based clustering, and visualization were performed using standard Seurat workflows with Uniform Manifold Approximation and Projection (UMAP) embedding. A clustering resolution of 0.1 was used to identify major leukocyte populations. Cells were clustered using Seurat’s graph-based clustering (FindNeighbors/FindClusters) with the default Louvain algorithm (algorithm=1) at resolution 0.1. To mitigate batch effects and technical variation, Harmony (V.1.2.3) was applied to Principal Component Analysis (PCA) embeddings, adjusting for donor, sequencing batch, and sex. NK cells were then subsetted and re-analyzed; 10 initial NK clusters were identified and subsequently curated and consolidated into 8 transcriptionally distinct NK cell subsets based on marker gene expression and annotation consistency.

Differentially expressed genes and cluster marker genes were identified using Seurat FindMarkers and FindAllMarkers, respectively, using the Wilcoxon rank-sum test. Genes were required to be expressed in at least 25% of cells in either group (min.pct=0.25) and to exhibit a minimum log2 fold-change of 0.25 (logfc.threshold=0.25).

P values were adjusted for multiple testing using the Bonferroni correction as implemented in Seurat, and genes with an adjusted p value<0.05 were considered statistically significant. Marker genes used for cell-type annotation are reported in the Results section.

Cell-type assignments were further validated using independent annotation resources, including Azimuth, CellMarker, and additional cell type–specific gene set libraries available through the Enrichr database.

### Flow cytometric characterization of NK cells and ILCs

NK cell surface markers were assessed using flow cytometry. The peripheral blood samples were collected from HDs and ascites-matched patients with OC. Buffy coats were prepared, and PBMCs were isolated by Ficoll density gradient centrifugation. In parallel, ascitic fluid was obtained from the patients with OC, and peritoneal cells were isolated using Lymphocyte Separation Medium 1077 (PromoCell) density gradient centrifugation. All ascites samples used for flow cytometry and scRNA-seq analyses were obtained from patients with histologically confirmed HGSOC and provided by the University Hospital of Marburg. Only treatment-naïve patients undergoing primary debulking surgery were included in the study. For flow cytometric analysis, cells were stained with fluorochrome-conjugated antibodies for 30 min at 4°C, followed by washing with fluorescence-activated cell sorting (FACS) buffer. Samples were acquired on a FACSCanto II Flow Cytometer and BD FACSCelesta Flow Cytometer (BD Biosciences, San Jose, California, USA). Cell-free ascites was obtained from the supernatant fraction and subjected to sequential centrifugation to remove residual cells and debris. Freshly isolated cell-free ascites was stored at −80°C and later used for in vitro NK cell culture experiments (figure 5).

For the analysis of ILCs by flow cytometry (Figure 6 and online supplemental figure S3F,G). Ascites samples were collected from a cohort of 16 patients with OC, stratified based on progression-free survival (PFS): 8 patients with high survival (PFS ≥24 months) and 8 patients with low survival (PFS ≤12 months). This classification enabled comparative profiling of ILC populations in relation to clinical outcome. PCs were isolated from ascitic fluid using Lymphocyte Separation Medium 1077 (PromoCell) density gradient centrifugation. For flow cytometric analysis, a similar procedure as used for NK cells was applied. A list of antibodies used for flow cytometry is provided in online supplemental table S1.

### NK cell culturing with ascites or medium

PBMCs from HDs were isolated from leucyte reduction systems by density gradient centrifugation. NK cells were subsequently purified from PBMCs using a negative selection NK cell isolation kit (Miltenyi Biotec), according to the manufacturer’s instructions. Cells were cultured in 12-well plates at a density of 1×10⁶ cells per well in Roswell Park Memorial Institute (RPMI) 1640 medium supplemented with GlutaMAX-I, 10% (v/v) fetal bovine serum, and 1% (v/v) penicillin/streptomycin, either alone or supplemented with 25% ascites fluid collected from a pooled sample of five patients.

For activated conditions, cells were stimulated with recombinant human IL-2 (200 U/mL; PeproTech, Cranbury, New Jersey, USA; Cat. #200-02-50) and recombinant human IL-15 (10 ng/mL; PeproTech; Cat. #200-15-10). For resting conditions, cells were cultured with low-dose recombinant human IL-2 (10 U/mL) for 24 hours. Following incubation, cells were harvested, and total RNA was isolated using the NucleoSpin RNA kit (Macherey-Nagel, Düren, Germany) according to the manufacturer’s protocol. cDNA synthesis was performed using the RevertAid First Strand cDNA Synthesis Kit (Thermo Fisher Scientific, Waltham, Massachusetts, USA).

For the experiments shown in figure 6D–F, the transforming growth factor (TGF)-β inhibitor SB-431542 (10 µM; Cayman Chemical/Biomol, Hamburg, Germany) was added to both conditions. Quantitative real-time PCR analyses were subsequently performed. Gene expression levels were normalized to the *RpL27* reference gene, and relative expression was calculated using the ΔΔCt method. Primer sequences are listed in online supplemental table S1. Additional experimental details are provided in the corresponding figure legends.

## Results

### Single-cell transcriptomic profiling unravels NK cell subset distribution and phenotype in OC ascites

**Figure 1:**
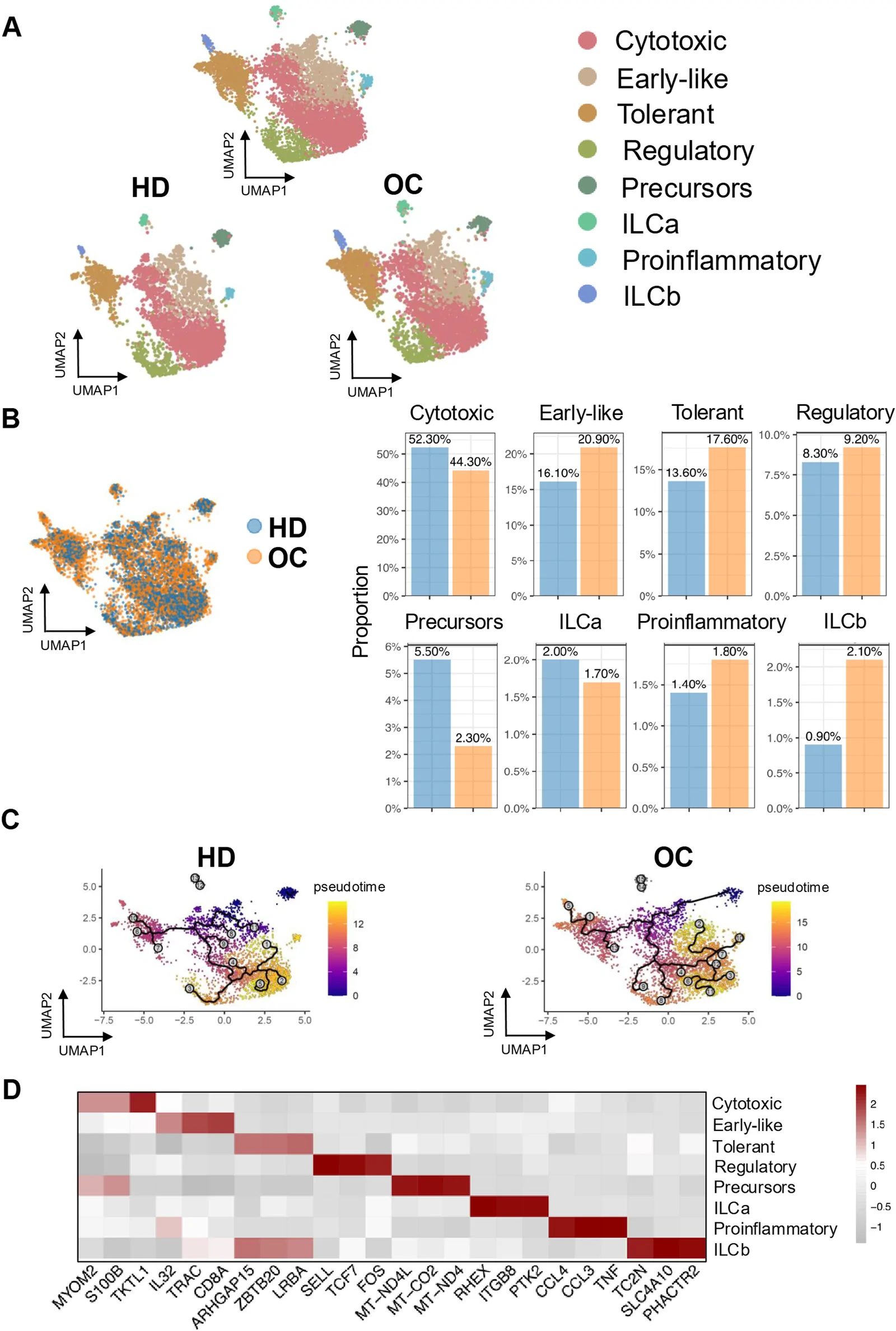
Single-cell RNA-seq analysis of enriched NK cells from peripheral blood of HD and OC ascites. (**A**) On the top UMAP visualization of NK cell subsets from ovarian cancer ascites (n=6) and healthy donor blood (n=3) after Harmony integration. Bottom UMAP plots show NK cell subsets in HD and OC samples separately. (**B**) Left: UMAP projection of NK-cell subsets from the integrated HD and OC datasets, highlighting shared and unique clusters between conditions. Right: Bar plots of the relative abundance of each NK-cell subset within total NK cells (percentage), shown separately for HD and OC. (**C**) Pseudotime trajectory analysis illustrating the inferred developmental progression of NK subsets, in HD- and OC-derived NK cells. (**D**) Heatmap showing the top three differentially expressed marker genes for each NK subset, regardless of conditions. HD, healthy donor; ILC, innate lymphoid cell; NK, natural killer; OC, ovarian cancer; scRNA-seq, single-cell RNA sequencing; UMAP, Uniform Manifold Approximation and Projection.

To elucidate the phenotype of NK cells within the OC microenvironment, specifically within the ascitic fluid, we performed scRNA-seq of CD3−CD56+NK cells (dataset^45^) isolated by negative selection from the ascites of patients with OC and peripheral blood of HDs (figure 1A). This analysis identified eight distinct NK subtype clusters reflecting the so far unknown heterogeneity of NK cells within the ascites. The clusters are characterized by the emergence of distinct functional states, including cytotoxic, early-like, tolerant, regulatory, precursors, proinflammatory, and innate lymphoid cells (figure 1A); some of these clusters were previously reported in other studies.^46^

Comparative analysis of cluster proportions was performed with Scillus package (R V.0.5.0), the analysis revealed differences in NK cell proportions between tumor-associated NK cells from ascites and HD-derived NK cells (figure 1B). Cytotoxic and precursor clusters were more abundant in peripheral HD NK cells, whereas the percentage of early-like, tolerant, regulatory, and proinflammatory clusters was higher in OC-derived NK cells. Two clusters of innate lymphoid cells were determined based on their expression profile and referred to as ILCa and ILCb. Pseudotime trajectory was performed separately for NK cells derived from OC-ascites and HDs, the data reflecting the progressive maturation of NK cells from less differentiated (purple), more detectable in HD-NK cells, to highly differentiated cytotoxic NK cells (yellow), more detectable in the OC-ascites NK cells (figure 1C).

The top three differentially expressed marker genes for each ILC subset implicate their different functions (figure 1D). The cytotoxic cluster upregulated myomesin-2, S100 calcium-binding protein B, and transketolase-like 1, while the early-like cluster expressed IL-32, T-cell receptor α constant, and CD8α molecule. The tolerant cluster showed higher Rho GTPase-activating protein 15, zinc finger and BTB domain-containing protein 20, and LPS-responsive beige-like anchor protein (LRBA), and the regulatory cluster upregulated L-selectin, transcription factor 7 (TCF7), and Fos proto-oncogene (FOS). The precursor cluster was enriched for mitochondrial genes MT-ND4L, MT-CO2, and MT-ND4. As expected, the proinflammatory cluster was enriched with cytokines, including C-C motif chemokine ligands 4 and 3 (CCL4 and CCL3) and TNF. The ILC subsets showed elevated levels of regulator of hemoglobinization and erythroid cell expansion, integrin β8, and protein tyrosine kinase 2 in ILCa, and upregulation of tandem C2 domains nuclear protein, solute carrier family 4 member 10, and phosphatase and actin regulator 2 in ILCb. Together, these markers delineate distinct NK phenotypes, providing a framework for immune-based characterization of the ascitic innate immune compartment.

### Cytotoxic and tolerance-associated NK cell states display distinct transcriptional and functional programs in patients with ascitic OC, with implications for immunotherapy responsiveness

**Figure 2:**
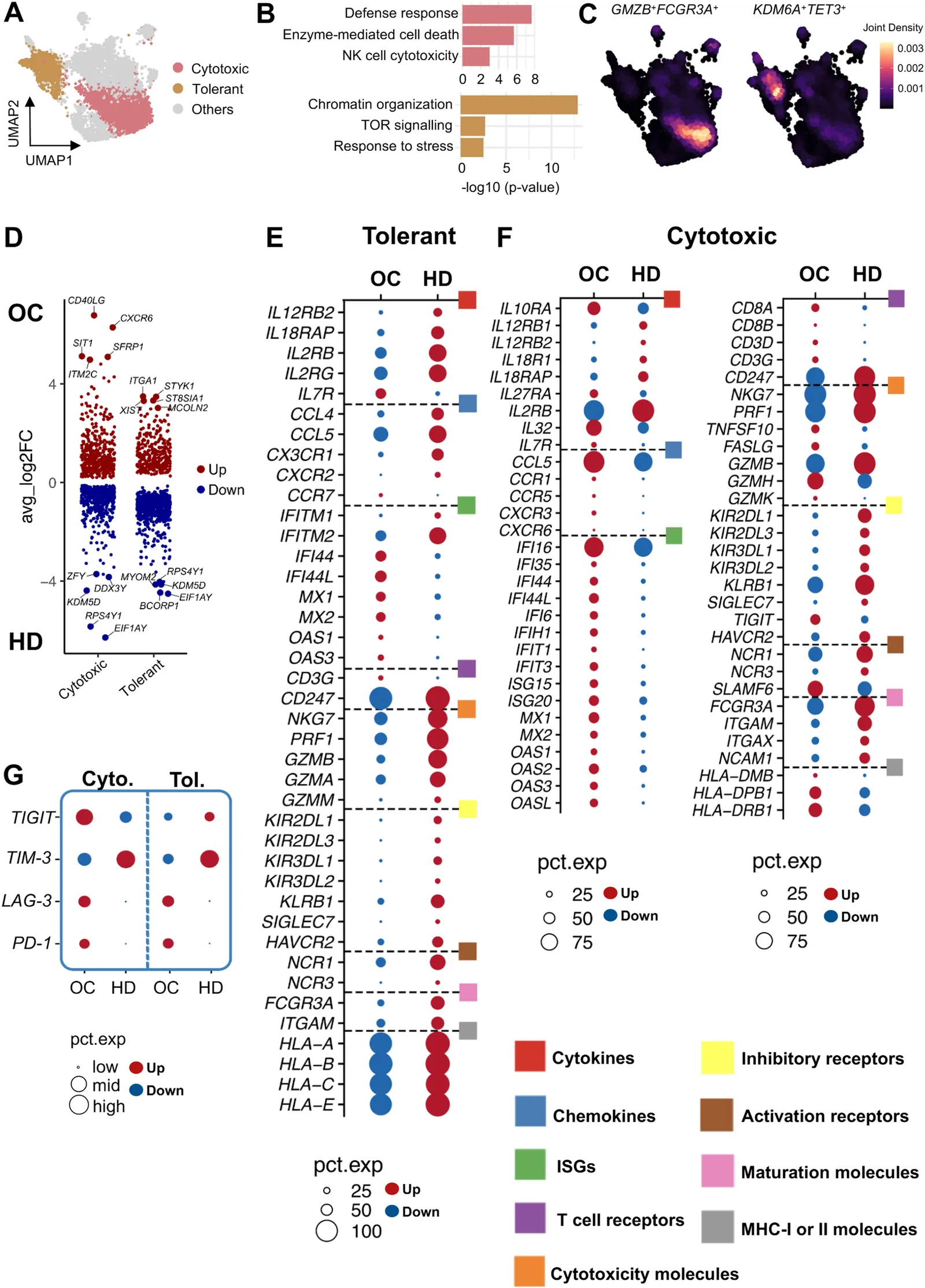
Inflammatory profiles of cytotoxic and tolerant NK cell subsets. (**A**) UMAP visualization of cytotoxic and tolerant NK cell subsets. (**B**) Bar plots showing the top three enriched biological pathways of expressed genes (DEGs) associated with cytotoxic and tolerant NK subsets using −log10 (p value). (**C**) Density plots showing expression distributions of two top-selected marker genes enriched within cytotoxic (*GMZB^+^FCGR3A^+^*) and tolerant (*KDM6A^+^TET3^+^*) NK subsets. (**D**) Scatter plot of DEGs between OC and HDs in cytotoxic and tolerant NK subsets. (**E, F**) Dot plots depicting expression patterns of inflammatory and functional NK-related genes between OC and HDs in tolerant (**E**) and cytotoxic (**F**) NK subsets. Dot color represents gene expression between OC and HDs NK cells, displayed as red (upregulated in HD-NKs) and blue (downregulated in OC-NKs). Dot size indicates the percentage of cells expressing each gene (pct.exp). (**G**) Dot plot showing expression levels of exhaustion-associated genes between OC and HD in tolerant and cytotoxic NK subsets. Dot color represents gene expression of selected exhaustion markers between OC- and HD-NK cells, red (upregulated) and blue (downregulated). Dot size indicates the percentage of cells expressing each gene (pct.exp). DEG, differentially expressed gene; FC, fold change; HD, healthy donor; LAG-3, lymphocyte-activation gene 3; MHC, major histocompatibility complex; NK, natural killer; OC, ovarian cancer; PD-1, programmed cell death protein 1; UMAP, Uniform Manifold Approximation; TIGIT, T-cell immunoreceptor Ig; TIM-3, T-cell immunoglobulin and mucin-domain containing-3; TOR, target of rapamycin.

We focused on the transcriptionally defined NK-cell clusters identified in figure 1, comprising a cytotoxic population and a tolerant, or immunoregulatory, population, as previously reported (figure 2A).^46^ To functionally validate this classification, we performed pathway enrichment analysis (figure 2B). Cytotoxic NK cells showed significant upregulation of pathways involved in defense response, enzyme-mediated cell death, and NK cell-mediated cytotoxicity, supporting their identity as terminal effector NK cells (figure 2B).^47 48^ On the other side, tolerant NK cells exhibited enrichment of pathways related to chromatin organization, cellular stress response, and the target of rapamycin signaling pathway. These pathways are commonly linked to differentiation and maturation of NK cells, with mTOR in particular, which is essential for NK cell development.^49 50^ Among the highest expressed genes in the cytotoxic NK cells cluster were granzyme B *(GZMB)* and Fc gamma receptor IIIa (*FCGR3A*, *CD16*) (figure 2C), both canonical markers of active, cytolytic NK cells.^23 51^ In contrast, the tolerant NK cluster upregulated lysine demethylase 6A (*KDM6A*), which is associated with reduced effector functions in female NK cells^52^ and Tet methylcytosine dioxygenase 3 (*TET3*) (figure 2C), a gene involved in chromatin remodeling and epigenetic regulation, and correlated with poor prognosis in patients with OC.^53^ Overall, the transcriptomic profiles of cytotoxic and tolerant NK cell clusters revealed distinct patterns of upregulated (red) and downregulated (blue) genes, reflecting substantial transcriptional differences between the subsets and also in comparison with HD-NK cells and patients with OC (figure 2D). This revealed the need for more refined strategies to dissect the activation and inhibitory programs operating within each cluster. To address this, we investigated key regulatory genes across both clusters, focusing on those involved in cytokine and chemokine signaling, cellular maturation, and receptor-ligand interactions (figure 2E,F).

Comparing OC and HD-derived cells, the OC-derived tolerant NK cells showed generally a reduced expression of genes essential for activation and effector functions (figure 2E,F), among them, for example, interleukin-2 receptor subunit beta (*IL2RB)* and interleukin-2 receptor subunit gamma *(IL2RG)*, which encode the β and γ subunits of the IL-2 receptor and are critical for NK cell proliferation and IL-2 responsiveness.^54 55^ Furthermore, key cytotoxic effectors, including *GZM*B along with the activation marker *CD247* (CD3ζ), were markedly downregulated in OC-tolerant NK cells, reflecting a transcriptional state indicative of impaired cytolytic potential.^56 57^ The downregulation of major histocompatibility complex (*MHC*) class I and II genes, including human leukocyte antigens *HLA-A* and *HLA-C* may suggest disrupted communication between OC-derived tolerant NK cells and other immune populations, such as T cells and dendritic cells.^58^

Similarly, the OC-derived cytotoxic NK cells exhibited marked downregulation of key effector genes (figure 2F) including *IL2RB*, *CD247*, *NKG7*, *PRF1*, *GZMB*, killer-cell immunoglobulin-like receptors (*KIRs*), killer cell lectin-like receptor subfamily B member 1, natural cytotoxicity triggering receptor 1, and *FCGR3A*, essential genes for NK activation, cytotoxic granule release, and ADCC,^56 57 59 60^ reflecting impaired functionality of cytotoxic OC-NK cells. Moreover, the cytotoxic OC-derived NK cells showed an upregulation of IL-10 receptor subunit alpha and inflammatory mediators such as *IL32*, *CCL5*, *GZMH*, as well as IFNG-inducible protein 16, which points to a tumor-driven reprogramming of NK cells. Different from the tolerant OC-derived NK cells, the OC-derived cytotoxic NK cells exhibited an increased expression of MHC class II genes (*HLA-DMB*, *DMB1*, *DRB1*), revealing different levels of regulation by immune cell interactions within the TME.^58^

We further investigated the phenotypic features associated with exhaustion-related markers in NK cells (figure 2G). The cytotoxic NK cells from patients with OC exhibited marked upregulation of exhaustion-associated markers, including T-cell immunoreceptor Ig (*TIGIT*), lymphocyte-activation gene 3 (*LAG-3*), and *PD-1*, compared with HD-derived cytotoxic NK cells. In addition, the tolerant OC-NK cells displayed a similar but more restricted pattern (figure 2G), characterized by elevated expression of *LAG-3* and *PD-1*. Of note, and unexpectedly, T-cell immunoglobulin and mucin-domain containing-3 (TIM-3) expression was diminished in both cytotoxic and tolerant NK cells. These findings support the presence of a phenotype associated with exhaustion-related markers across both cytotoxic and tolerant NK cell populations within the ascitic TME.

### Patients with OC-derived ascitic CD56^+^CD16^+^ NK cell subsets impair cytotoxic function

**Figure 3:**
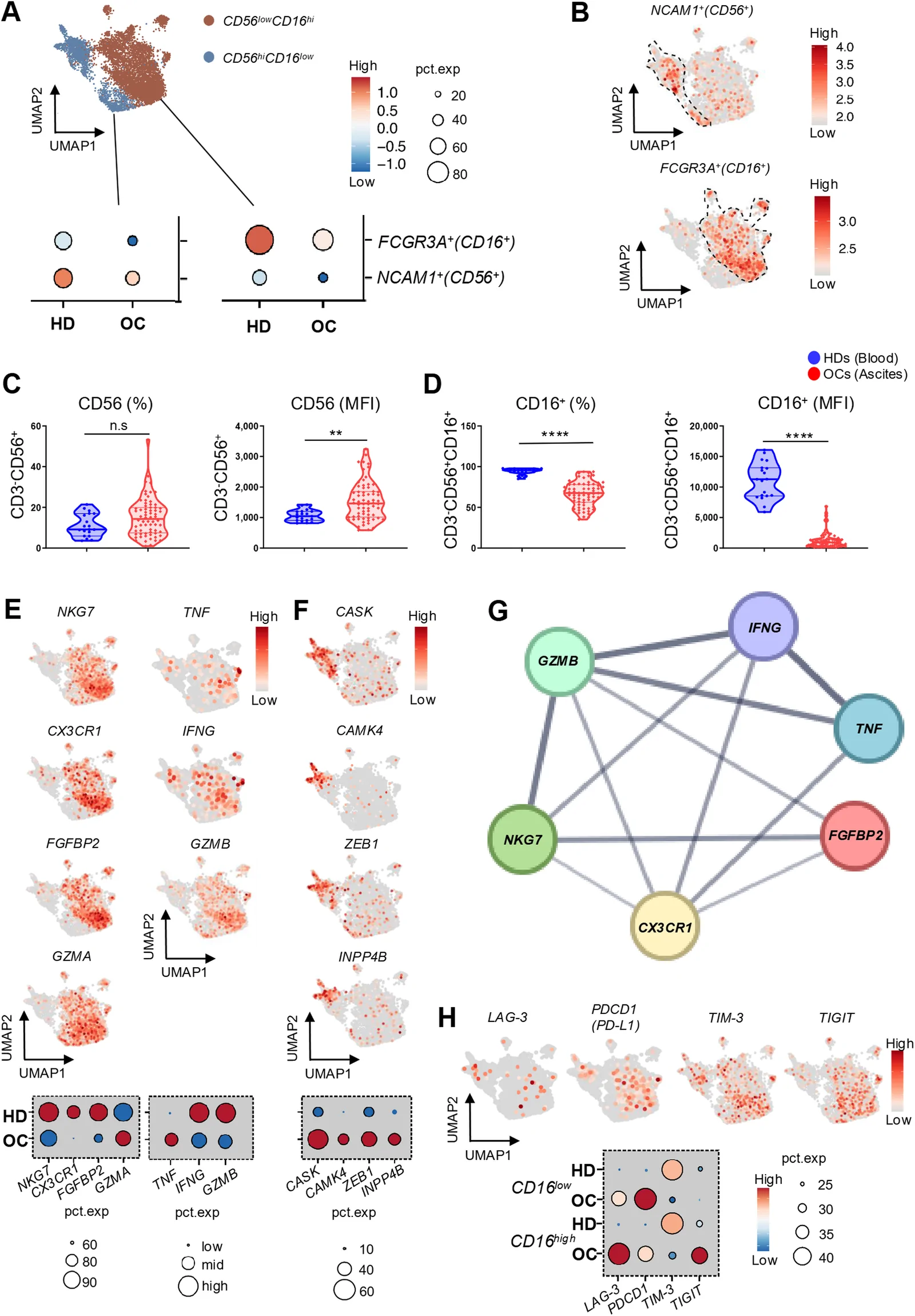
Subclustering of NK cell subsets based on CD16 and CD56 expression. (**A**) UMAP visualization of CD56^high^CD16^low^ and CD56^low^CD16^high^ NK cell subclusters, with feature plots showing the expression patterns of CD16 (FCGR3A) and CD56 (NCAM1). pct.exp: percentage of cells expression. (**B**) UMAP plot illustrating the expression of CD16 (FCGR3A) and CD56 (NCAM1) in CD56^high^CD16^low^ and CD56^low^CD16^high^ NK cell subclusters, independent of NK cells source. (**C, D**) Violin plots showing flow cytometric analysis of CD56^+^CD3⁻ and CD56^+^CD16^+^CD3⁻ NK cell populations from the peripheral blood of HDs (blue) and ascitic fluid of patients with OC (red). The percentage and MFI of each population are shown. Each symbol represents an individual biological replicate (mean±SEM; n=19–68). Statistical significance was assessed using unpaired t-tests. **p<0.01; ****p<0.0001. (**E**) UMAP and feature plots of the top upregulated genes (*NKG7*, *CX3CR1*, *FGFBP2*, and *GZMA*) and inflammatory genes (*TNF*, *IFNG*, and *GZMB*) enriched in CD56^low^CD16^high^ NK cells, with a dot plot showing the percentage of cells expressing (pct.exp) of these genes in NK cells comparing HDs-NKs with ascites OC-NK cells; red (upregulated) and blue (downregulated). (**F**) Feature plots of the top upregulated genes (*CASK*, *CAMK4*, *ZEB1*, and *INPP4B*) enriched in CD56^high^CD16^low^ NK cells, with a dot plot showing the percentage of cells expressing (pct.exp) of these genes in NK cells comparing HDs-NK with ascites NK cells. (**G**) STRING protein–protein interaction network analysis (https://string-db.org) of the genes shown in figure 3E. (**H**) Feature plots of selected exhaustion markers (*LAG-3*, *PDCD1* (PD-1), *TIM-3*, and *TIGIT*) enriched in NK cells, with a dot plot showing the percentage of cells expression (pct.exp) of these genes in CD16^high^ and CD16^low^ NK cells comparing HDs-NK with ascites NK cells. HD, healthy donor; IFN, interferon; LAG-3, lymphocyte-activation gene 3; MFI, mean fluorescence intensity; NK, natural killer; ns, not significant; OC, ovarian cancer; PD-1, programmed cell death protein 1; PD-L1, programmed death-ligand 1; TIGIT, T-cell immunoreceptor Ig; TIM-3, T-cell immunoglobulin and mucin-domain containing-3; TNF, tumor necrosis factor.

Next, we stratified NK cell populations based on canonical surface markers CD56 and CD16 using single-cell transcriptomic data (figure 3A,B). This analysis revealed a substantial shift in subset distribution, with OC-derived NK cells enriched in CD56^high^CD16^low^ (immunoregulatory) populations and depleted in CD56^low^CD16^high^ subsets (cytotoxic)^61^ (figure 3A).

To validate these transcriptomic findings at the protein level, we performed flow cytometric analysis of NK cells from HDs’ peripheral blood (PBMCs) and ascitic fluid/PCs of patients with OC (figure 3C,D). Consistent with scRNA-seq data, NK cell frequency (CD56^+^CD3⁻) was slightly higher and the mean fluorescence intensity (MFI) of CD56 was significantly increased in ascites-derived NK cells (figure 3C), further supporting a phenotypic shift toward a CD56^high^ and immunoregulatory phenotype.^61^ Moreover, the frequency of CD56^+^CD16^+^CD3⁻ NK cells was significantly reduced within ascitic fluid compared with HDs’ blood (figure 3D), a pattern mirrored by reduced CD16 surface expression (MFI) in the ascitic NK population (figure 3D). Analysis of surface receptor expression on ascites-derived NK cells compared with matched patient peripheral blood NK cells and healthy donor peripheral blood NK cells revealed that ascites-derived NK cells display distinct phenotypic features, suggesting a local impact of the ascitic microenvironment (online supplemental figure S1A–E). Consistent with their reduced CD16 expression (online supplemental figure S1A), ascites-derived NK cells showed reduced overall as well as reduced CD16-dependent killing activity compared with HD-NK cells (online supplemental figure S2A).

These findings are consistent with our previous report describing an altered NK cell composition in OC ascites, with enrichment of CD56^high^/CD16^low^ subsets associated with reduced cytotoxicity.^62^

Focusing on the CD56^low^CD16^high^ NK subset typically associated with cytotoxic function,^61 63^ we observed that this subset was diminished in OC-NK cells, and in comparison, to HD-NK cells characterized by the marked downregulation of genes including *NKG7*, fibroblast growth factor binding protein 2 (*FGFBP2*), and *CX3CR1*, whereas *GZMA* was higher expressed (figure 3E). Of note, the cytotoxicity-related genes (*GZMB, IFNG*) in CD56^low^CD16^high^ patient NK cells were significantly downregulated and *TNF* expression showed a modest increase (figure 3E). Notably, these genes have been reported to be associated with distinct CD4 T-cell phenotypes. NKG7, FGFBP2, and CX3CR1 are characteristic of terminally differentiated cytotoxic CD4 T cells,^64^ while all of these genes have also been linked to precursor populations of cytotoxic CD4 T cells.^65^

On the other hand, immunoregulatory CD56^high^CD16^low^ NK subset, which is enriched in ascitic NK cells,^61 63^ displays among the top upregulated genes calcium/calmodulin-dependent serine protein kinase*,* calcium/calmodulin-dependent protein kinase IV*,* zinc finger E-box binding homeobox 1*,* and inositol polyphosphate-4-phosphatase type II B (figure 3F), these factors so far not described in the context of NK cell function.

STRING analysis revealed that several genes upregulated in the CD56^low^CD16^high^ NK subset of HDs were strongly connected to each other within the protein–protein interaction network (figure 3G).

Moreover, the expression of exhaustion markers *LAG-3* and *TIGIT*, and, to a lesser extent PD-1 (*PDCD1*) was higher in the CD16^high^ subset, while CD16^low^ NK cells showed pronounced PD-1 expression and modest LAG-3 upregulation (figure 3H). These findings suggest that OC-NK cells exhibit phenotypic features associated with exhaustion-related marker expression.

### NK cells isolated from OC ascites display diminished activity and elevated exhaustion-related markers compared with HD peripheral NK cells

**Figure 4:**
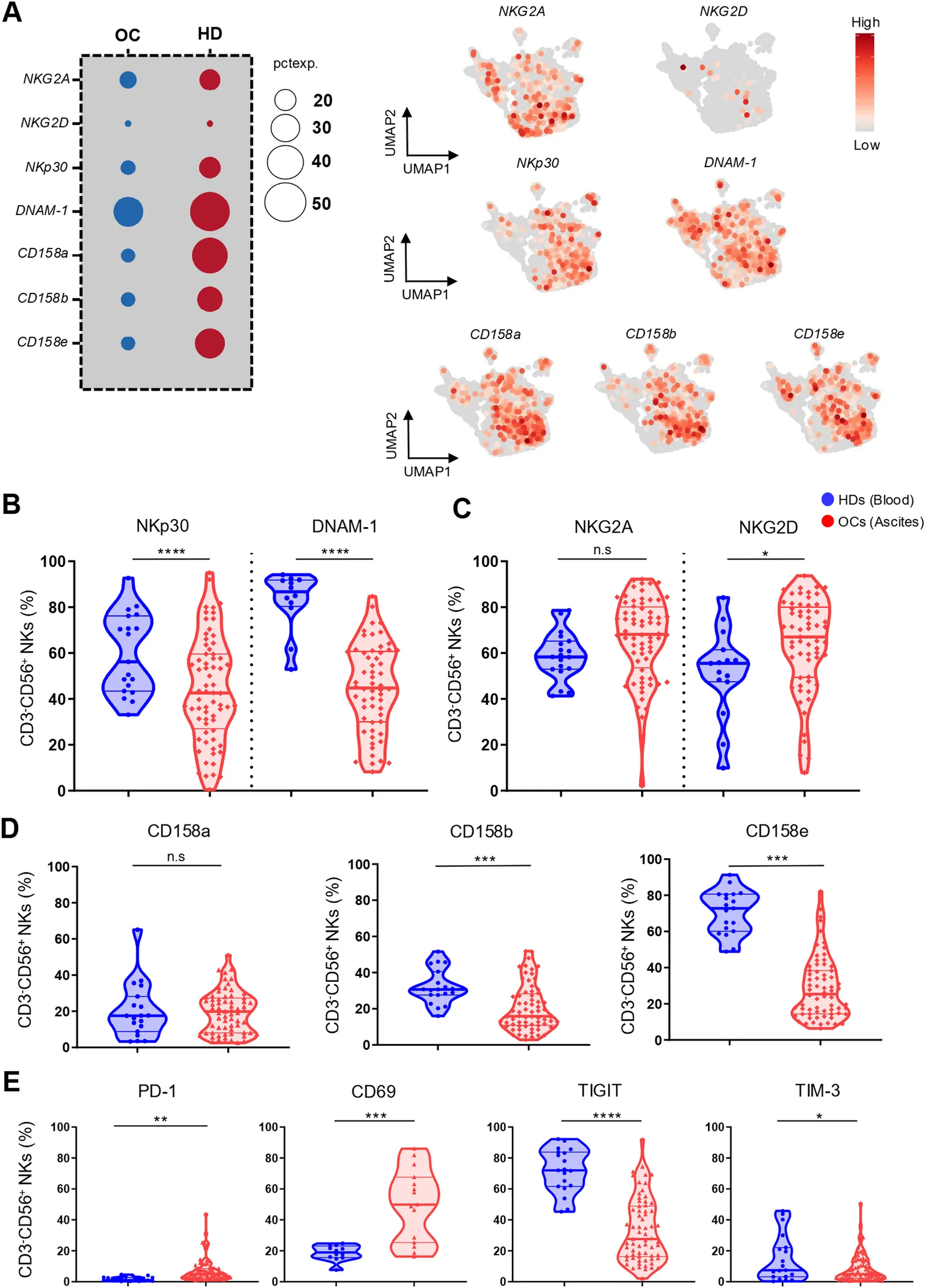
NK cells from OC ascites exhibit reduced activity and an exhaustion-related phenotype. (**A**) Left: Dot plot showing the percentage of cells expression (pct.exp) patterns of surface markers associated with NK cell activation and inhibition, including *NKG2A*, *NKG2D*, *NKp30* (*NCR3*), *DNAM-1* (*CD226*), *CD158a*, *CD158b*, and *CD158e*, in NK cells from ascitic fluid of patients with OC and peripheral blood of HDs. Right: Feature plots depicting the distribution of these genes across NK cell subclusters. pct.exp: percentage of cell expression; red (upregulated) and blue (downregulated). (**B**–**D**) Violin plots showing flow cytometric analysis of the percentages of selected activation and inhibitory proteins in CD56^+^CD3⁻ NK cells from the peripheral blood of HDs (blue) and ascites of patients with OC (red). Each symbol represents an individual biological replicate (mean±SEM; n=19–68). (**E**) Violin plots showing flow cytometric analysis of the percentages of selected exhaustion markers in CD56^+^CD3⁻ NK cells from the peripheral blood of HDs and ascites of patients with OC. Each symbol represents an individual biological replicate (mean±SEM; n=19–68). Statistical significance was assessed using unpaired t-tests. *p<0.05; **p<0.01; ***p<0.001; ****p<0.0001. HD, healthy donor; NK, natural killer; ns, not significant; OC, ovarian cancer; PD-1, programmed cell death protein 1; TIGIT, T-cell immunoreceptor Ig; TIM-3, T-cell immunoglobulin and mucin-domain containing-3.

Given the compromised cytotoxic and enriched tolerant NK scRNA-based cluster in patient-derived cells, we analyzed the expression profile of important surface markers associated with activation, inhibition, or exhaustion-related markers of NK cells. The scRNA-seq demonstrated downregulation of *NKG2A*, *NKG2D*, *NKp30* (NCR3), *DNAM-1* (CD226), *CD158a*, *CD158b*, and *CD158e* in OC-derived NK cells compared with HD peripheral NK cells (figure 4A). This was in line with a diminished surface expression for selected receptors expressed on OC NK cells isolated from ascitic fluid compared with blood-derived HD-NK cells. Additionally, we evaluated the surface expression of activating and inhibitory receptors, including NKG2A, NKG2D, NKp30, DNAM-1, KIRs (CD158a, CD158b, and CD158e), and activating receptor CD69, as well as the selected exhaustion-related markers PD-1, TIGIT, and TIM-3 on CD3^−^CD56^+^ lymphocytes (figure 4B–E). The OC-NK cells demonstrated significant downregulation of the key activating receptors NKp30 and DNAM-1 compared with HD-NK cells in blood (figure 4B), whereas NKG2A was unchanged on the protein level and NKG2D was significantly upregulated in OC-NK cells (figure 4C) as described earlier.^66^

Moreover, a significant downregulation of CD158b (KIR2DL2/3) and CD158e (KIR3DL1) was observed in OC-NK cells isolated from ascitic fluid compared with peripheral blood-derived HD-NK cells (figure 4D). Consistent with our scRNA-seq findings, this reduction was evident across both cytotoxic and tolerant NK cell subsets in OC samples (figure 2E,F). Notably, the expression of CD158a (KIR2DL1) remained unchanged between HDs and OC groups in our cohort (figure 4D).

The exhaustion-related marker PD-1, and activating marker CD69 were significantly upregulated in OC-NK cells isolated from ascitic fluid compared with peripheral blood-derived HDs-NK cells (figure 4E). This pattern was consistent with our single-cell transcriptomic data, where both cytotoxic and tolerant OC-NK cell clusters exhibited elevated PD-1 expression relative to HD-NK cells (figure 2E–G). Interestingly, TIGIT and TIM-3 were unexpectedly downregulated in ascites-derived OC-NK cells compared with HD-NK cells (figure 4E). This discrepancy with the transcriptional upregulation observed in the scRNA-seq data likely reflects context-dependent regulation, and may include post-transcriptional and post-translational mechanisms, as well as dynamic modulation of receptor surface expression.

We further analyzed the same surface markers associated with NK cell activation, inhibition, and exhaustion in matched peripheral blood OC-NK cells (online supplemental figure S1B–E). Comparison of matched peripheral blood OC-NK cells with ascites-derived OC-NK cells revealed alterations, including decreased expression of DNAM-1 and CD158e, whereas NKG2D, NKG2A and CD69 expression were increased (online supplemental figure S1B–D). The exhaustion-related marker PD-1 showed a similar expression pattern in matched peripheral blood OC-NK cells and HD-NK cells (online supplemental figure S1E), whereas CD69 was downregulated in matched peripheral blood OC-NK cells compared with both HD-NK and ascites-derived OC-NK cells (online supplemental figure S1E). TIGIT and TIM-3 were both significantly downregulated in ascites-derived OC-NK cells and matched peripheral blood OC-NK cells compared with HD-NK cells (online supplemental figure S1E). Together, these findings suggest that the ascites compartment exerts additional local effects beyond systemic immune alterations.

### Ascites significantly contributes to NK cell impairment and diminished cytotoxic activity

**Figure 5:**
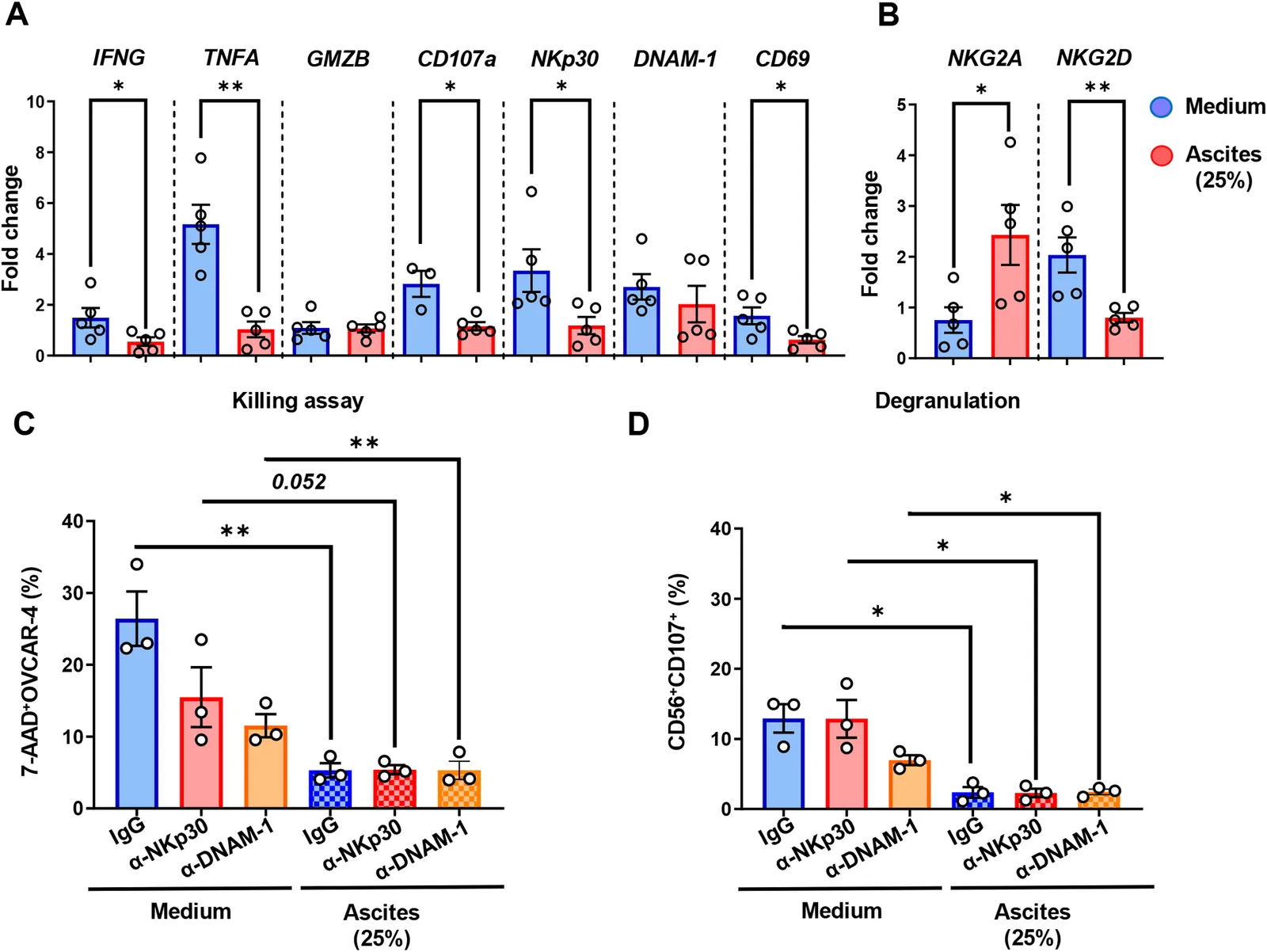
Impact of ascites on HD-NK cell activity. (**A, B**) Gene expression analysis of NK cells isolated from the peripheral blood of HDs and cultured in 24-well plates in RPMI medium alone or supplemented with 25% ascites fluid pooled from five patients with OC. Cells were incubated for 24 hours at 37°C, followed by total RNA isolation and cDNA synthesis. qRT-PCR was performed to assess gene expression levels. Data are presented as fold change relative to control conditions and normalized to the *RpL27* reference gene (mean±SEM; n=5, except for CD107a, n=3). Statistical significance was assessed using paired t-tests. *p<0.05; **p<0.01. (**C**) Cytotoxic activity of HD effector PBLs against OVCAR-4 target cells. Effector PBLs were generated as described in the Methods section and were then pre-incubated in 6-well plates (10×10^6^ per well) with 10 µg/mL of isotype control IgG (BioLegend, Koblenz, Germany; MOPC-21; 400102), anti-NKp30 (BioLegend, Koblenz, Germany; p30-15; Cat: 325204), or anti-DNAM-1 (Miltenyi Biotec, Bergisch Gladbach, Germany; DX11; Cat: 130–092-479) blocking antibodies for 30 min at room temperature. Effector cells, that is, PBLs, were then co-cultured with CellTracker (Violet BMQC (Invitrogen))-labeled OVCAR-4 target cells in round-bottom 96-well plates at an effector-to-target cell ratio of 100:1, either in medium alone or supplemented with 25% ascites fluid. After incubation, target cell death was quantified by flow cytometric analysis of 7-AAD^+^OVCAR-4^+^ cells (mean±SEM; n=3). Statistical significance was assessed using paired t-tests. *p<0.05. (**D**) Percentage of CD56^+^CD107a^+^ cells among effector PBLs treated with the same procedure as in (**C**). Anti-CD107a–PE antibody and 2 µM monensin (BioLegend) were subsequently added, followed by an additional 4 hours of incubation. Cells were analyzed by flow cytometry (mean±SEM; n=3). Statistical significance was assessed using paired t-tests. *p<0.05. cDNA, complementary DNA; HD, healthy donor; NK, natural killer; OC, ovarian cancer; PBL, peripheral blood lymphocyte.

To determine whether the ascitic microenvironment drives the functional defects observed in OC-derived NK cells, we performed in vitro experiments in which NK cells isolated from HDs were cultured for 24 hours in either standard media or ascites fluid. NK cells exposed to ascites showed a marked reduction in the expression of *IFNG*, *TNFA*, *CD107A*, *NKp30*, and *CD69* relative to media-cultured NK cells (figure 5A). Unexpectedly, the expression of *GZMB* and *DNAM-1* remained unchanged (figure 5A). In addition, ascites-cultured NK cells displayed increased expression of *NKG2A* and reduced expression of *NKG2D* (figure 5B), consistent with an inhibitory shift in receptor balance.

We further analyzed the killing activity of ascites-NK cells (figure 5C,D). Here, the NK cells derived from HDs were pre-incubated with blocking antibodies against the activating receptors NKp30 or DNAM-1, demonstrating that killing of OVCAR-4 cells was at least partly dependent on these receptors (figure 5C). Of note, both killing activity (figure 5C) and degranulation were diminished in the presence of ascites (figure 5D), demonstrating that ascites treatment induced a global reduction in NK cells activity independent of the activating receptors blocking such as NKp30 or DNAM-1.

Collectively, these findings demonstrate that short-term exposure to ascites is sufficient to impair NK-cell activity, supporting the conclusion that ascitic factors contribute directly to the dysfunctional phenotype observed in OC-associated NK cells, and identifying soluble suppressive programs that may be therapeutically targetable.

### Ovarian cancer ascites promotes type 2 innate immune skewing through ILC2 accumulation and TGF-β-mediated NK-cell dysfunction

**Figure 6:**
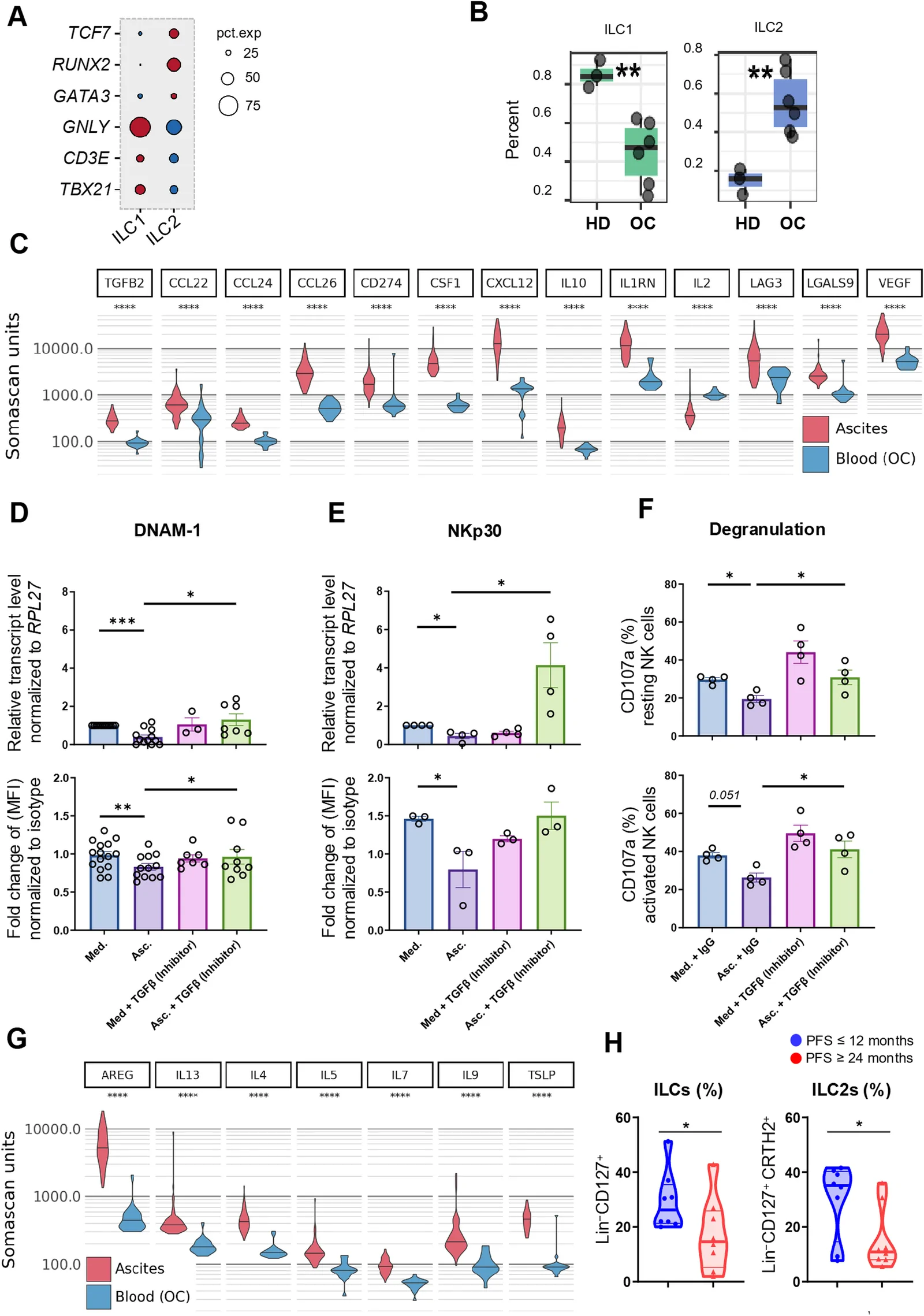
Ovarian cancer ascites drives type 2 innate immunity via ILC2 accumulation and TGF-β-dependent NK-cell dysfunction. (**A**) Dot plot illustrating the percentage of cells expression (pct.exp) patterns of established marker genes between the two clusters of ILCs to annotate the ILC1 and ILC2 subsets, red (upregulated), blue (downregulated). (**B**) Bar plots depicting their relative abundance (percentage) in OC-ascites and HD peripheral blood. (**C**) SOMAScan proteomic analysis of selected proteins associated with NK cell activation and inhibitory signals in ascites (OC-ascites=70 and OC-plasma=20). Violin plot, central bar denotes median. Statistical significance was assessed using unpaired t-tests on log2-transformed data. *p<0.05; **p<0.01; ***p<0.001; ****p<0.0001; red (ascites OC) and blue (matched-blood OC). (**D, E**) Relative mRNA expression of *DNAM-1* and *NKp30*, normalized to the *RpL27* reference gene, and fold change in MFI of the corresponding surface proteins in NK cells isolated from HDs. NK cells were cultured for 24 hours in medium alone or supplemented with 25% ascites fluid, in the presence or absence of a TGF-β receptor inhibitor (10 µM). (**F**) Percentage of CD56^+^CD107a^+^ NK cells isolated from the peripheral blood of HDs (mean±SEM; n=4). NK cells were cultured under resting or activated conditions, as described in the Methods section, and subsequently incubated for 24 hours in medium alone or supplemented with 25% ascites, with or without TGF-β inhibition (10 µM). CD107a expression was assessed by flow cytometry. (**G**) SOMAScan proteomic analysis of selected proteins associated with cytokine production and functional enhancement in ILCs (OC-ascites=70 and OC-plasma=20). Violin plot, central bar denotes median. Statistical significance was assessed using unpaired t-tests on log2-transformed data. *p<0.05; **p<0.01; ***p<0.001; ****p<0.0001; red (ascites OC) and blue (matched-blood OC). (**H**) Violin plots show frequencies of Lin⁻CD127^+^ cells (total ILCs) and Lin⁻CD127^+^CRTH2^+^ cells (ILC2s) in ascites samples from patients with OC stratified by (PFS (≤12 months, blue, n=8; PFS ≥24 months, red, n=8); blue (shorter survival patients with OC) and red (longer survival patients with OC). For panels (**D, E, F and H**), data are presented as mean±SEM. Statistical significance was assessed using paired t-tests; *p<0.05; **p<0.01; ***p<0.001. HD, healthy donor; ILC, innate lymphoid cell; MFI, mean fluorescence intensity; mRNA, messenger RNA; NK, natural killer; ns, not significant; OC, ovarian cancer; PFS, progression-free survival; TGF, transforming growth factor.

scRNA-seq analysis revealed clear segregation of ILC subsets in OC ascites. Given their functional overlap with NK cells, ILCs were analyzed as part of the innate immune compartment.^67^ We first established a gating strategy to focus on ILCs (online supplemental figure S2C). Then the CD200 receptor 1 was used to distinguish ILCs from NK cells (online supplemental figure S3A), identifying two transcriptionally distinct clusters (figure 6A and online supplemental figure S3B). ILC1s expressed T-box transcription factor 21 and *GNLY*, consistent with type 1 effector functions, whereas ILC2s showed high expression of *GATA3*, Runt-related transcription factor 2, and *TCF7*, in line with established type 2 ILC programs^68^ (figure 6A).

Compared with HDs, patients with OC displayed a significant reduction in ILC1 frequency and a marked expansion of ILC2s in ascites (figure 6B). Gene Set Enrichment Analysis of cytotoxicity and exhaustion scores was curated with SeuratExtend package (R V.1.0.0), the data demonstrated a strong upregulation of exhaustion signatures in OC-ILC2s relative to HD-ILC2s, while only minor changes were observed in ILC1s (online supplemental figure S3C). Cytotoxicity-associated gene signatures were significantly reduced in both OC-ILC1s and OC-ILC2s compared with their HD counterparts (online supplemental figure S3C), indicating broad functional impairment.

Differential gene expression analysis of ILC2s revealed significant downregulation of genes linked to immune activation and favorable prognosis, including *CD74*, ferritin heavy chain 1 (*FTH1*), ubiquinol-cytochrome c reductase hinge protein, tropomyosin 4 (*TPM4*), and *FOS* (online supplemental figure S3D,E). These genes play important roles in antigen presentation and immunotherapy responsiveness, for example, *CD74*,^69 70^ oxidative stress regulation (*FTH1*),^71^ and cell proliferation, apoptosis, and migration control (*FOS*),^72^ collectively indicating suppressed ILC2 effector function in OC ascites.

To identify potential microenvironmental drivers, SOMAscan proteomic profiling compared OC ascites with matched OC plasma.^73^ Ascites exhibited a strongly immunosuppressive signature (figure 6C) with elevated TGF-β 2, C-C motif chemokine ligand 22 (CCL22), CCL24, CCL26, programmed death-ligand 1 (PD-L1/CD274), colony-stimulating factor 1 (CSF1), CXCL12, IL10, IL1RN, LAG3, galectin 9, and vascular endothelial growth factor, all associated with NK cell inhibition and immune evasion (figure 6C). In contrast, IL-2 levels were significantly reduced in ascitic fluid (figure 6C).

Given the abundance of TGF-β and its known effects on NK cell receptors, NK cells from HDs were cultured in OC ascites with or without TGF-β inhibition. Ascites exposure significantly reduced DNAM-1 and NKp30 expression at both transcript and protein levels, while TGF-β blockade partly restored their expression, although to a lesser extent for DNAM-1 (figure 6D,E). Functionally, ascites suppressed CD107a degranulation in both resting and cytokine-activated NK cells, whereas TGF-β inhibition rescued degranulation, particularly under ascitic conditions (figure 6F). To determine whether the suppressive effects of ascites are patient-specific, activated HD-NK cells were cultured with TGF-β-high (≥2,459 arbitrary units) or TGF-β-low (<2,459 arbitrary units) ascites based on the mean SOMAScan TGFB1 level, with or without an anti-TGF-β antibody. TGF-β-high ascites induced stronger suppression of activating NK cell receptors at the protein level (NKp30, NKG2D, CD16, and CD69), whereas TGF-β blockade partially restored their expression (online supplemental figure S2B), suggesting that TGF-β contributes to the suppressive effects of ascites.

In parallel, OC ascites was enriched in cytokines and alarmins associated with ILC2 differentiation and activation, including amphiregulin (AREG), IL-13, IL-4, IL-5, IL-7, IL-9, and thymic stromal lymphopoietin (TSLP), compared with OC plasma (figure 6G), providing a mechanistic basis for ILC2 accumulation.

Clinically, patients with poor prognosis with PFS (≤12 months) exhibited significantly higher frequencies of total ILCs and ILC2s in ascites than patients with favorable outcomes (PFS ≥24 months) (figure 6H), a pattern also reflected by increased geometric MFI (online supplemental figure S3F). ILC2s from patients with short PFS expressed higher levels of exhaustion-associated marker PD-1, whereas ILC2s from patients with long PFS showed increased GZMB expression (online supplemental figure S3G), suggesting a potential immunoregulatory role of PD-1^+^ ILC2s in ascites. Importantly, infiltrating PD-1^+^ NK cells and CD127^+^GATA3^+^PD-1^+^ ILC2s were also detected in primary HGSOC tissues, demonstrating that these immune cell populations are present in both the primary tumor tissue and malignant ascites (online supplemental figure S4A,B).

Together, these findings suggest that OC ascites is characterized by a type 2-polarized, immunosuppressive microenvironment that promotes the accumulation of PD-1^+^ ILC2s and impairs NK cell function, thereby linking immune regulation within the TME to adverse clinical outcomes.

## Discussion

This study provides a comprehensive single-cell atlas of innate lymphocytes in patients with OC ascites, integrating scRNA-seq with extensive phenotypic and functional analyses of NK cells and ILCs, and positions these innate immune states within a translational framework relevant to immunotherapy development. Our study demonstrates that OC ascites establishes a coordinated, cytokine-driven immune signaling milieu that profoundly reshapes innate immune cells. This environment, which provides TGF-β and type 2 alarmins, leads to a marked depletion of mature CD56^low^CD16^high^ cytotoxic NK cells, expansion of early-like and tolerant NK populations, and accumulation of PD-1^+^ ILC2s. Importantly, the functional state of these cells correlates with PFS, highlighting a clinically relevant link between innate immune remodeling and OC trajectory. Together, these results define OC ascites not as a passive fluid compartment but as a potent immunoregulatory niche that actively diminishes innate antitumor immunity.

Malignant ascites is a key contributor to the immunosuppressive TME in OC, where it impairs the antitumor activity of immune cells, including NK and T cells.^74 75^ Our scRNA-seq data demonstrate a striking shift in NK cell composition in ascites compared with HD peripheral blood, characterized by reduced cytotoxic clusters and increased early-like, tolerant, and regulatory NK states. This shift appears to be driven by soluble factors within the ascitic fluid, which have previously been shown to impair NK and T-cell cytotoxicity and function.^40 74 76 77^ Pseudotime trajectory analysis further demonstrated a developmental blockade at early-to-intermediate stages, consistent with impaired NK cell maturation and reduced effector potential. These data confirm and extend previous reports on TME-induced NK cell dysfunction, including ascitic fluid, reported in OC and other malignancies.^78–83^ Furthermore, the transcriptional identity of NK cell clusters, defined by the expression of key upregulated genes, underscores the robustness and reproducibility of our mapping.^46 68 84 85^

Functionally, our data show that both cytotoxic and tolerant NK cell subsets are reprogrammed by the OC ascitic TME when compared with NK cells from HDs. Cytotoxic NK cells exhibited reduced expression of key effector genes, PRF1, GZMB, NKG7, NKp30 (*NCR3*), and CD226 (DNAM-1), all essential for activation and cytotoxicity.^86–93^ In parallel, tolerant NK cells upregulated immunoregulatory and epigenetic regulators such as KDM6A and TET3, consistent with a suppressive and metabolically reprogrammed phenotype. Flow cytometric validation confirmed increased protein expression of PD-1 and LAG-3 across both subsets, with TIGIT selectively elevated in cytotoxic NK cells.^94–98^ Functional assays demonstrated that ascites exposure impaired degranulation and reduced cytotoxicity against ovarian tumor targets, partly mediated by NKp30 and DNAM-1. NKp30, a critical activating receptor that binds B7-H6 and BAG6, is central to NK cell-mediated tumor recognition; its downregulation has been linked to poor prognosis in OC and melanoma.^90 91^ Additionally, the engagement of DNAM-1 ligand and receptor on NK cells leads to enhanced DNAM-1-mediated NK cell lysis in OC,^43^ and upregulation of both receptors, NKp30 and DNAM-1, is associated with the activation of NK cells in ovarian neoplasia.^44^ These findings indicate that ascites-driven loss of NK effector function is associated with transcriptional reprogramming and changes in receptor expression patterns. Targeting these dysfunctional pathways may offer therapeutic strategies to restore NK cell activity in OC.

To uncover the soluble mediators responsible for this dysfunction, we integrated SOMAscan proteomics. Proteomic profiling revealed high levels of TGF-β, PD-L1 (CD274), CSF1, CXCL12, and immunosuppressive chemokines (CCL22, CCL24, CCL26), alongside elevated IL-10 and reduced IL-2, which is an essential cytokine for NK maintenance in ascites. Exposure of HD-derived NK cells to ascitic fluid recapitulated the impaired phenotype, including reduced *IFNG*, *TNFA*, *CD107a*, *NKp30*, and *CD69*, and altered expression of *NKG2* family receptors. Strikingly, pharmacologic inhibition of TGF-β restored NKp30 and DNAM-1 levels and rescued CD107a degranulation even in ascites, establishing the contribution of TGF-β in NK dysfunction in patients with OC. This aligns with known functions of TGF-β as a suppressor of NK activity.^78 99^ Thus, TGF-β blockade can partially overcome ascites-induced NK cell impairment and enhance innate antitumor activity in vitro. However, the incomplete rescue indicates that additional factors within the ascitic microenvironment contribute to NK cell dysfunction and warrant further investigation.

Ascites-derived NK cells displayed high CD56 and significantly reduced CD16 expression compared with HDs, suggesting a less mature and less cytotoxic phenotype, as reported in various cancers.^100 101^ Supporting this, scRNA-seq revealed that CD56^low^CD16^high^ NK cells from HDs expressed higher levels of effector genes such as *NKG7*, *FGFBP2*, and *CX3CR1*, which were markedly downregulated in OC ascitic NK cells.^102 103^ While *GZMA* and *TNF* were modestly elevated, this likely reflects ongoing inflammation rather than active cytotoxicity. Both CD16^high^ and CD16^low^ NK cell subsets from ascites showed increased expression of exhaustion-related markers, with CD16^high^ cells displaying particularly strong *PD-1*, *LAG-3*, and *TIGIT* expression. This likely reflects the dynamic nature of NK cell receptor regulation in the TME and is in line with previous data demonstrating that NK cell dysfunction in HGSOC is driven by ascitic fluid.^3^ Here, the inhibitory activity could be attributed to lipid uptake and metabolic changes, mechanisms that might act in concert with cytokine-dependent effects.

As expected, NKG2A was upregulated ex vivo on ascites treated HD-NK cells. Impaired function of NK cells was associated with high level inhibitory receptor NKG2A.^104^ Furthermore, KIR expression was selectively altered, with CD158b and CD158e significantly downregulated in ascitic NK cells. Given the role of KIRs in HLA-mediated activation, such modulations may block NK cell responsiveness and cytotoxicity in the OC TME.^105^ In summary, the OC ascitic TME reshapes NK cells toward a less mature and dysfunctional phenotype.

In addition to NK cells, our study provides a characterization of ILC subtypes in OC ascites. ILCs are increasingly recognized as important regulators of antitumor immunity.^27 106–108^ We observed reduced ILC1s, which are functionally analogous to NK cells and often protective in cancer,^27^ and enrichment of ILC2s, which have been associated with protumor functions in several malignancies.^109^ This shift is consistent with ILC plasticity and its responsiveness to the cytokine milieu.^110^ Ascites contained high levels of IL-4, IL-5, IL-9, IL-13, IL-7, TSLP, and AREG, all canonical drivers of ILC2 differentiation.^111^ Previous reports described context-dependent, either tumor-promoting or suppressive roles of ILC2s in different cancers.^112^ Notably, ILC2s from ascites exhibited decreased cytotoxic score and increased exhaustion score. Key downregulated genes included *CD74*, implicated in antigen presentation in lung cancer ILC2s,^113^ and *FTH1* and *TPM4*, which exert tumor-suppressive roles in multiple cancers.^114 115^ Together, these findings indicate that ascites fosters the accumulation of PD-1^+^ ILC2s. Further studies are required to better define the role of ILC2s in shaping the TME and their role in tumor progression and therapy responses.

Clinical relevance emerged from the correlation between ILC2 abundance and phenotype with patient outcome. Patients with shorter PFS exhibited higher ILC2 frequencies and increased PD-1 expression, whereas patients with longer PFS had higher GZMB expression in ILCs. These observations agree with reports of context-dependent ILC2 roles: protective in melanoma and colorectal cancer,^116 117^ yet tumor-promoting in lung, breast, and gastric cancers, and acute promyelocytic leukemia.^30 32 118 119^ Our findings suggest that in OC, PD-1^+^ ILC2s expansion may contribute to disease progression.

## Conclusion

Ascites-derived mediators, including TGF-β and type 2 cytokines, collectively suppress innate lymphocytes as NK cells undergo functional impairment, characterized by reduced activating receptor expression and loss of cytotoxicity, whereas ILC2s expand but adopt dysfunctional PD-1^high^GZMB^low^ states. At the same time, cytotoxic NK subsets and ILC1s, both known to exert antitumor effector functions, are depleted. Despite limitations related to the small cohort size, our integrated single-cell, proteomic, and functional analyses provide a detailed framework for understanding ascites-driven innate immune suppression in OC and nominate ascites-associated NK/ILC states as candidates for immunotherapeutic targeting.

## Supplementary material

10.1136/jitc-2026-015396 online supplemental table 1

10.1136/jitc-2026-015396 online supplemental file 1

## Data Availability

Data are available in a public, open access repository. Data are available upon reasonable request.
