# Supplementary material for: Ascites reprograms innate lymphoid immune cells in ovarian cancer by promoting ILC2 enrichment and dysfunctional NK-cell states
Source: J Immunother Cancer. 2026 Aug 26;14(8):e015396. doi: 10.1136/jitc-2026-015396 (PMC13536108; doi:10.1136/jitc-2026-015396)
Supplement: Supplementary file 1 [file jitc-14-8-s002.pdf]

Supplementary File

Ascites reprograms innate lymphoid immune cells in ovarian cancer by promoting ILC2 enrichment and dysfunctional NK-cell states

Bilal Alashkar Alhamwe[1#], Fahd Alhamdan[2#], Viviane Ponath[1#], Nathalie Hoffmann[1], Celina Meena[1], Florian Fingernagel[1,3], Holger Garn[4], Kim Pauk[4], Corinna Keber[5], Carsten Denkert[5], Silke Reinartz[6], Elke Pogge von Strandmann[1,7*]

[1]Marburg University, Institute for Tumor Immunology, Marburg, Germany
[2]Department of Anesthesiology, Critical Care, and Pain Medicine, Cardiac Anesthesia Division, Boston Children's Hospital, Boston, USA; Department of Immunology and Anaesthesia, Harvard Medical School, Boston, MA, USA.
[3]Marburg University, Core facility Genomics, Marburg, Germany.
[4]Marburg University, Translational Inflammation Research Division & Core Facility for Single Cell Multiomics, Medical Faculty, Marburg, Germany
[5]Marburg University and University Hospital Marburg (UKGM), Institute of Pathology, Marburg, Germany
[6]Marburg University, Translational Oncology, Center for Tumor Biology and Immunology (ZTI), Marburg, Germany
[7]Marburg University, Core Facility Extracellular Vesicles EV-iTEC, Marburg, Germany

[#]Bilal Alashkar Alhamwe, Fahd Alhamdan, and Viviane Ponath contributed equally to this article.

*Corresponding author
Prof. Dr. Elke Pogge von Strandmann
Institute of Tumor Immunology
Center for Tumor and Immunobiology (ZTI)
Philipps University of Marburg
Hans-Meerwein-Str. 3
35043 Marburg, Germany
Tel.: +49 (0)6421 28-21640

Supplementary File

Methods

SomaScan-based proteomic profiling of ascites and plasma

Somascan for ascites and plasma samples from ovarian carcinoma patients has been previously published in Finkernagel et al (1). Survival analysis (Kaplain-Maier, logrank tests) were performed using the python lifelines package, version 0.30.0 (https://doi.org/10.21105/joss.01317).

Bioinformatic analysis

Gene ontology (GO) enrichment analysis was performed using clusterProfiler (v4.12.6) on cluster-specific upregulated marker genes (adjusted $p < 0.05$, avg_log2FC > 0.25) with GO Biological Process with signifcance cut off FDR<0.05. Protein–protein interaction network analysis was performed using STRING (STRING v12.0; Homo sapiens) on selected proteins. Interactions were filtered using a minimum required interaction score of ≥0.4 (medium confidence).

Degranulation and Killing Assay

OVCAR-4 cells were cultured in RPMI 1640 medium supplemented with GlutaMAX-I, 10% (v/v) fetal bovine serum (FBS), and 1% (v/v) penicillin/streptomycin (P/S). Cells were passaged two to three times per week depending on growth, and detached using trypsin on the day of the experiment.

Peripheral blood mononuclear cells (PBMCs) were purified by ficoll density centrifugation using LSM1077 (Sigma-Aldrich). PBMCs were incubated in 6-well plates for 2 hours then the non-adherent Peripheral blood lymphocytes (PBLs) were collected. The effector PBLs was generated by treating PBLs with 10 µg/mL of either isotype control IgG, anti-NKp30 or anti-DNAM-1 blocking antibody for 30 minutes at room temperature. This followed by culturing pre-treated PBLs in 24-well plates for 24 hours either in complete medium (RPMI 1640 + GlutaMAX-I + 10% FBS + 1% P/S) or in 25% ascitic fluid pooled from five OC patients. The rIL-2 (10 U/mL) was added to support lymphocyte survival.

NK cells were isolated using the negative selection NK cell isolation kit (Miltenyi Biotec, Bergisch Gladbach, Germany). For the killing assay, the OVCAR-4 target cells were labeled with 5 µM CellTracker™ Violet BMQC (Invitrogen) in serum-free medium for 45 minutes at 37 °C, followed by two washes with complete medium. Effector NK cells and target (OVCAR-4) cells were co-cultured in round-bottom 96-well plates at an effector-to-target (E:T) ratio as indicated. Plates were centrifuged at 100 × g for 1 minute to promote cell contact and incubated for 3 hours at 37 °C. After incubation, cells were stained with 7-AAD (50 ng/well) to assess target cell death and analyzed by flow cytometry using a BD FACSCanto II (BD Biosciences).

For the degranulation assay, we followed same procedures. Effector and target tracked (OVCAR-4) cells were co-cultured in round-bottom 96-well plates and plates were centrifuged at 100 × g for 1 minute to promote cell contact and incubated for 2 hours at 37 °C. Then, 400 ng CD107a-PE and 2 µM Monensin (Biolegend) per well were added to the samples, followed by four hours of co-cultivation at 37 °C. Next, cells were washed with FACS buffer and stained with 60 ng CD56-BV421 for 30 minutes at 4 °C. The cells were washed with FACS buffer and analyzed on the FACS Canto II cytometer (BD Bioscience). For degranulation assay with TGFß blocking, cells were treated with (10 µg/mL) of either isotype control IgG or of TGF-β antibody or with the TGFß inhibitor (10 µM). CD107a (%) was tested in both activating and resting conditions by flow cytometry using a BD FACSCanto II (BD Biosciences).

Immunohistochemistry of human OC primary tumor tissue

A human FFP blocks (n=5) were cut into 2 µm sections and mounted on SuperFrost Plus slides for immunohistochemical staining. For immunohistochemistry staining of tissue sections from OC patients, a heat-induced epitope retrieval with EDTA was applied. Staining was done using a Bond III for GATA3 and a Dako Autostainer Link 48 for all other stainings. After effectively blocking endogenous peroxidase activity, sections were incubated for 45 minutes with mouse monoclonal anti-CD56 antibody (1:50, Agilent, #M7304), mouse monoclonal anti-PD-1 antibody (1:150, Zytomed, #516-18664), rabbit monoclonal anti-CD127 antibody (1:500, abcam, #EPR23747-333), and mouse anti-GATA3 antibody (1:50; Zytomed, #MSK100-05). Following this step, sections were washed and treated with Leica Bond Polymer Refine Detection for GATA3 and Dako REAL EnVision HRP Rabbit/Mouse polymer for all other stainings, designed to react with DAB chromogen, in accordance with the manufacturer's protocol. All stained tissues were scanned, and the analysis of digitized histology and staining results was performed using the advanced QuPath bioimaging analysis software (version 0.3.2).

Statistics

All statistical methods are described in the respective figure legends

References

1: Finkernagel F, Reinartz S, Schuldner M, Malz A, Jansen JM, Wagner U, Worzfeld T, Graumann J, von Strandmann EP, Müller R. Dual-platform affinity proteomics identifies links between the recurrence of ovarian carcinoma and proteins released into the tumor microenvironment. Theranostics. 2019 Aug 22;9(22):6601-6617. doi: 10.7150/thno.37549. PMID: 31588238; PMCID: PMC6771240.

2: Hao Y, Hao S, Andersen-Nissen E, Mauck WM 3rd, Zheng S, Butler A, Lee MJ, Wilk AJ, Darby C, Zager M, Hoffman P, Stoeckius M, Papalexi E, Mimitou EP, Jain J, Srivastava A, Stuart T, Fleming LM, Yeung B, Rogers AJ, McElrath JM, Blish CA, Gottardo R, Smibert P, Satija R. Integrated analysis of multimodal single-cell data. Cell. 2021 Jun 24;184(13):3573-3587.e29. doi: 10.1016/j.cell.2021.04.048. Epub 2021 May 31. PMID: 34062119; PMCID: PMC8238499.

3: Hu C, Li T, Xu Y, Zhang X, Li F, Bai J, Chen J, Jiang W, Yang K, Ou Q, Li X, Wang P, Zhang Y. CellMarker 2.0: an updated database of manually curated cell markers in human/mouse and web tools based on scRNA-seq data. Nucleic Acids Res. 2023 Jan 6;51(D1):D870-D876. doi: 10.1093/nar/gkac947. PMID: 36300619; PMCID: PMC9825416.

Supplementary File

SUPPLEMENTARY FIGURES and LEGENDS

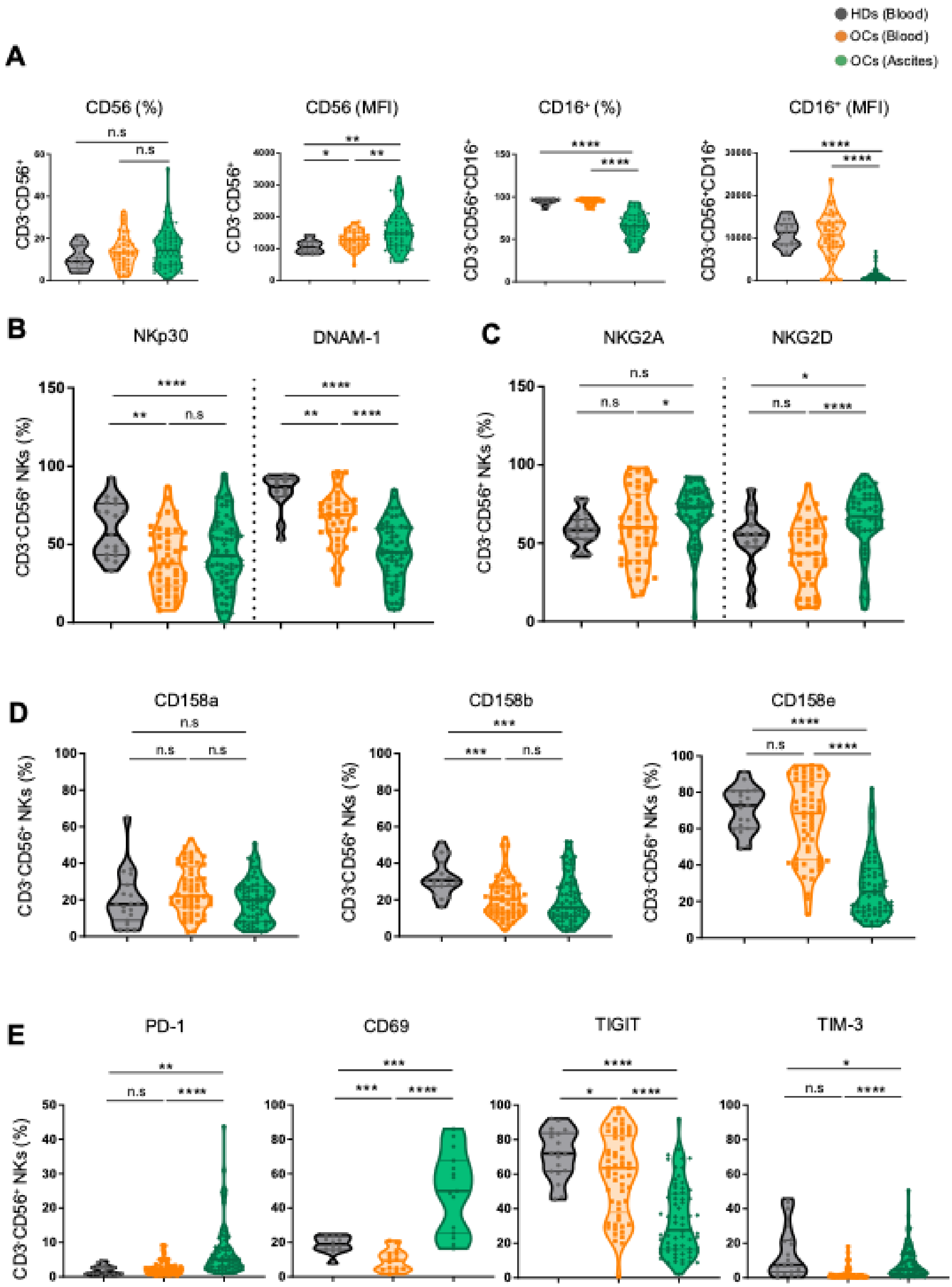

Supplementary Figure S1. NK activating and inhibitory surface markers expression. **(A, B)** Violin plots showing flow cytometric analysis of CD56⁺CD3⁻ and CD56⁺CD16⁺CD3⁻ NK cell populations from the

peripheral blood of HDs (black) and ascitic fluid of OC patients (green) and their available matched OC patients' peripheral blood (orange). The percentage and mean fluorescence intensity (MFI) of each population are shown. Each symbol represents an individual biological replicate (mean ± SEM; n = 19–68). Statistical significance was assessed using unpaired *t*-tests. *P < 0.05; **P < 0.01; ****P < 0.0001; n.s., not significant. **(B–D)** Violin plots showing flow cytometric analysis of the percentages of selected activation and inhibitory proteins in $CD56^+CD3^-$ NK cells from the peripheral blood of HDs (black) and ascitic fluid of OC patients (green) and their available matched OC patients' peripheral blood (orange). Each symbol represents an individual biological replicate (mean ± SEM; n = 19–68). Statistical significance was assessed using unpaired *t*-tests. *P < 0.05; **P < 0.01; ***P < 0.001; ****P < 0.0001; n.s., not significant. **(E)** Violin plots showing flow cytometric analysis of the percentages of selected exhaustion markers in $CD56^+CD3^-$ NK cells from the peripheral blood of HDs (black) and ascitic fluid of OC patients (green) and their available matched OC patients' peripheral blood (orange). Each symbol represents an individual biological replicate (mean ± SEM; n = 19–68). significance was assessed using unpaired *t*-tests. *P < 0.05; **P < 0.01; ***P < 0.001; ****P < 0.0001; n.s., not significant.

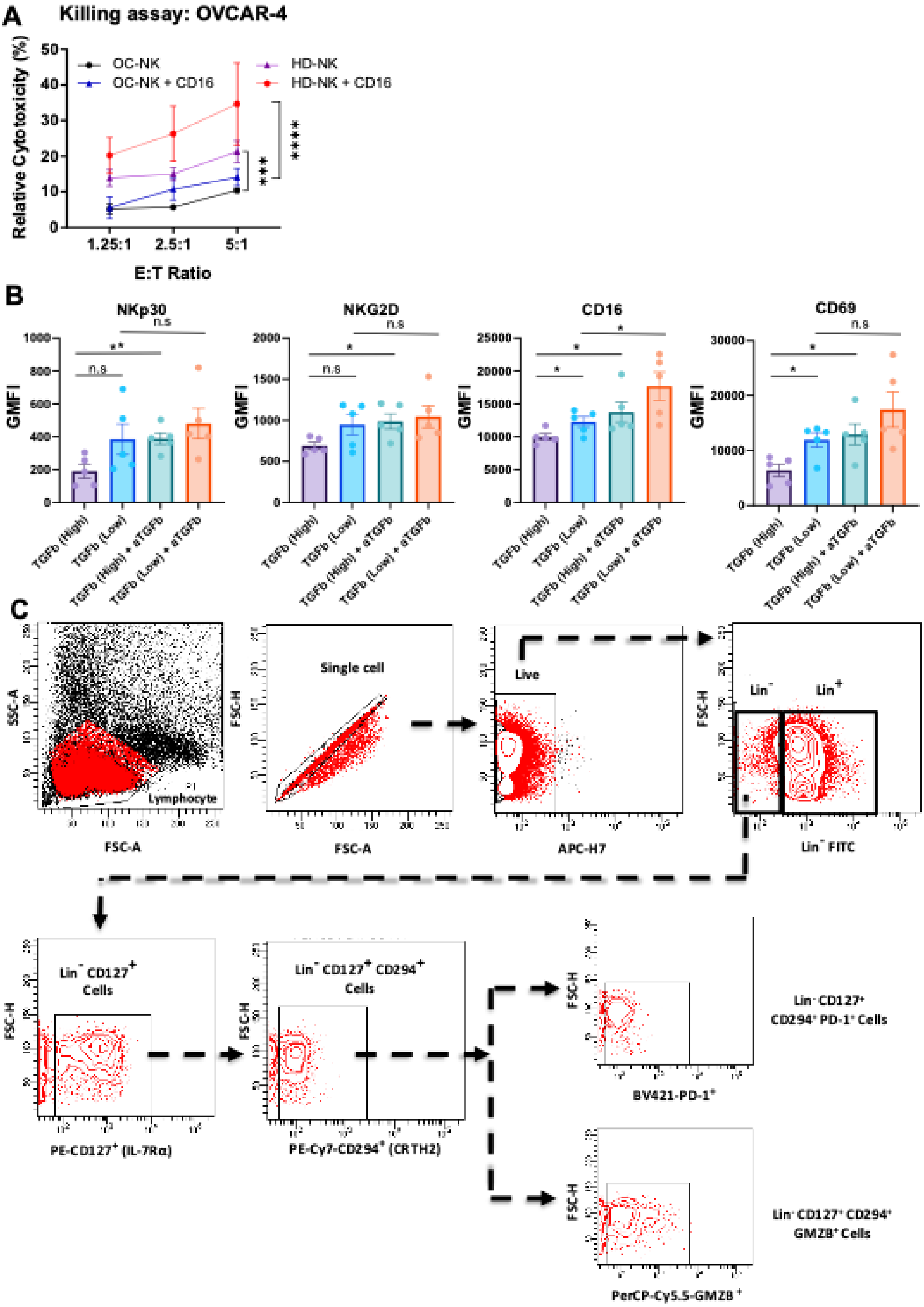

Supplementary Figure S2. CD16-mediated cytotoxicity, modulation of NK-cell phenotype by TGF-β, and gating strategy for ILC2 analysis. (A) Killing assay: Freshly isolated HD-derived NK cells or OC ascites-derived NK cells (n = 3 donors each) were co-cultured with CellTracker Violet BMQC-labeled

OVCAR-4 target cells at effector-to-target (E:T) ratios of 1.25:1, 2.5:1, and 5:1 for 3 h with or without prior CD16 activation. NK cells were added for 1 hour to anti-CD16 coated wells (clone 3G8, 1–2 µg/mL) for CD16-activation. Relative cytotoxicity means percentage of 7-AAD$^+$OVCAR-4$^+$ cells representing dead target cells and was quantified by 7-AAD staining using flow cytometry. Statistical significance was assessed using two way anova. **(B)** Geometric mean fluorescence intensity (GMFI) of NKp30, NKG2D, CD16, and CD69 on HD-derived NK cells following pre-stimulation with rhIL-2 (200 U/mL) for 24 h and subsequent incubation for 24 h with ovarian cancer ascites containing high or low levels of TGF-β, with or without anti-TGF-β antibody (10 µg/mL). TGF-β-high (n=5) and TGF-β-low ascites (n=5) were defined using the mean SomaScan TGFB1 level (2459 arbU) as the cut-off. **(C)** Gating strategy for the identification of ILC2s from ovarian cancer ascites. Peritoneal cells were gated sequentially on singlets, live lymphocytes, Lineage$^-$CD127$^+$ cells, followed by CD294 (CRTH2), PD-1, and granzyme B expression as indicated.

Supplementary File

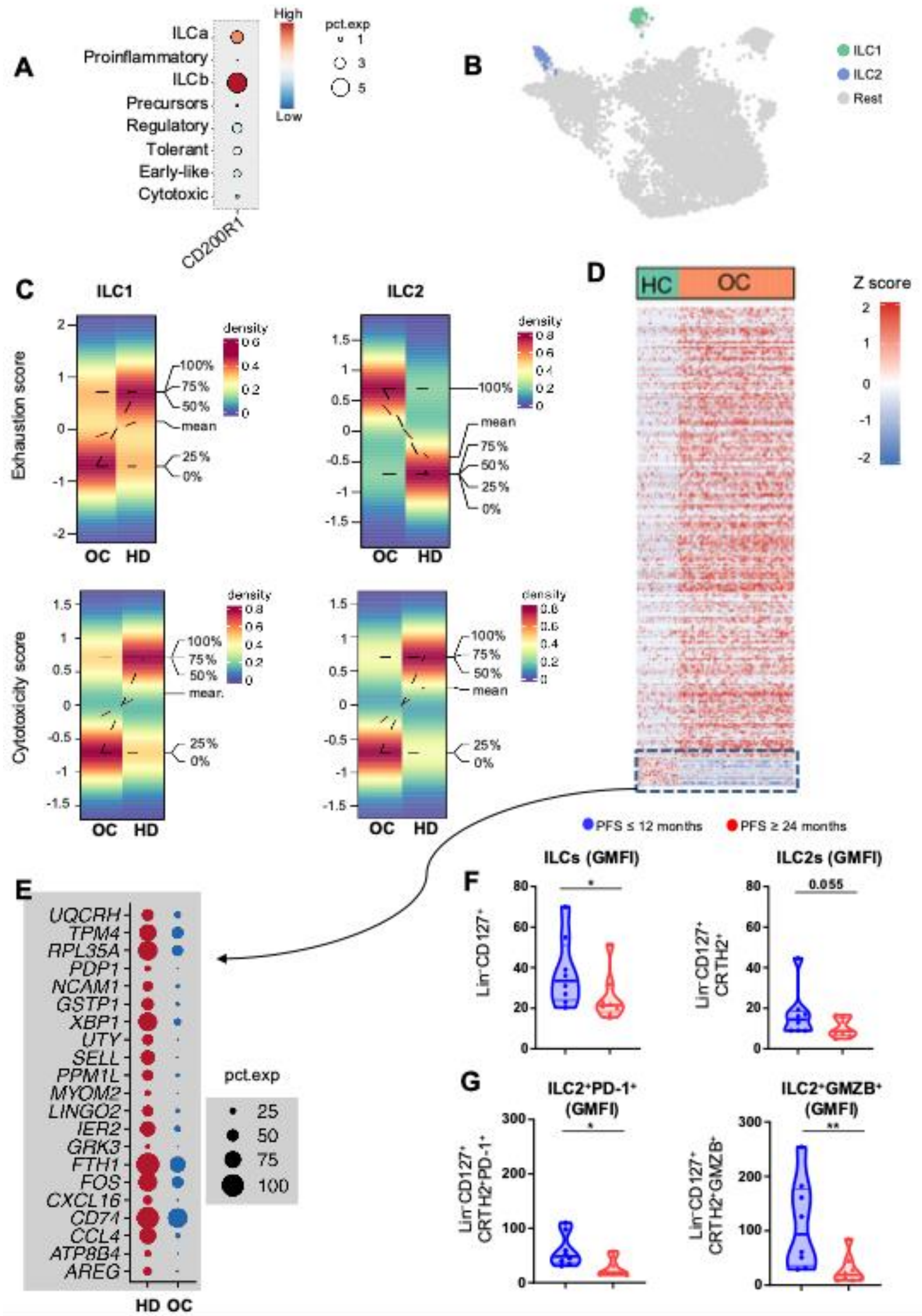

Supplementary Figure S3. Exhausted ILC2s accumulate in ovarian cancer ascites.

(A) Dot plot showing the percentage of expression (pct.exp) of the *CD200R1* gene across NK cell clusters to annotate the ILCs subsets.

(B) UMAP visualization of ILC1 (green) and ILC2 (blue) clusters.
(C) Upper panel: Density heatmap showing exhaustion scores in ILC1 and ILC2 subsets in OC and HD samples. Lower panel: Density heatmap showing cytotoxicity scores in ILC1 and ILC2 subsets in OC and HD samples.
(D) Heatmap displaying differentially expressed genes (DEGs) in ILC2 cells comparing OC and HD samples.
(E) Dot plot showing the percentage of expression (pct.exp) patterns of ILC2-associated genes that are downregulated in OC (Blue) relative to HD (Red).
(F) Violin plots represent geometric mean fluorescence intensity (GMFI) of Lin^-CD127^+ cells (total ILCs) and $Lin^-CD127^+CRTH2^+$ cells (ILC2s) in ascites samples from ovarian cancer (OC) patients stratified by progression-free survival (PFS ≤ 12 months, blue, n = 8; PFS ≥ 24 months, red, n = 8).
(G) Violin plots represent flow cytometric analysis of $Lin^-CD127^+CRTH2^+PD\text{-}1^+$ ILC2s and $Lin^-CD127^+CRTH2^+GZMB^+$ ILC2s in ascites samples.
For panels **(F, G)**, data are presented as mean ± SEM. Statistical significance was assessed using paired *t*-tests. *P < 0.05; **P < 0.01; n.s., not significant.

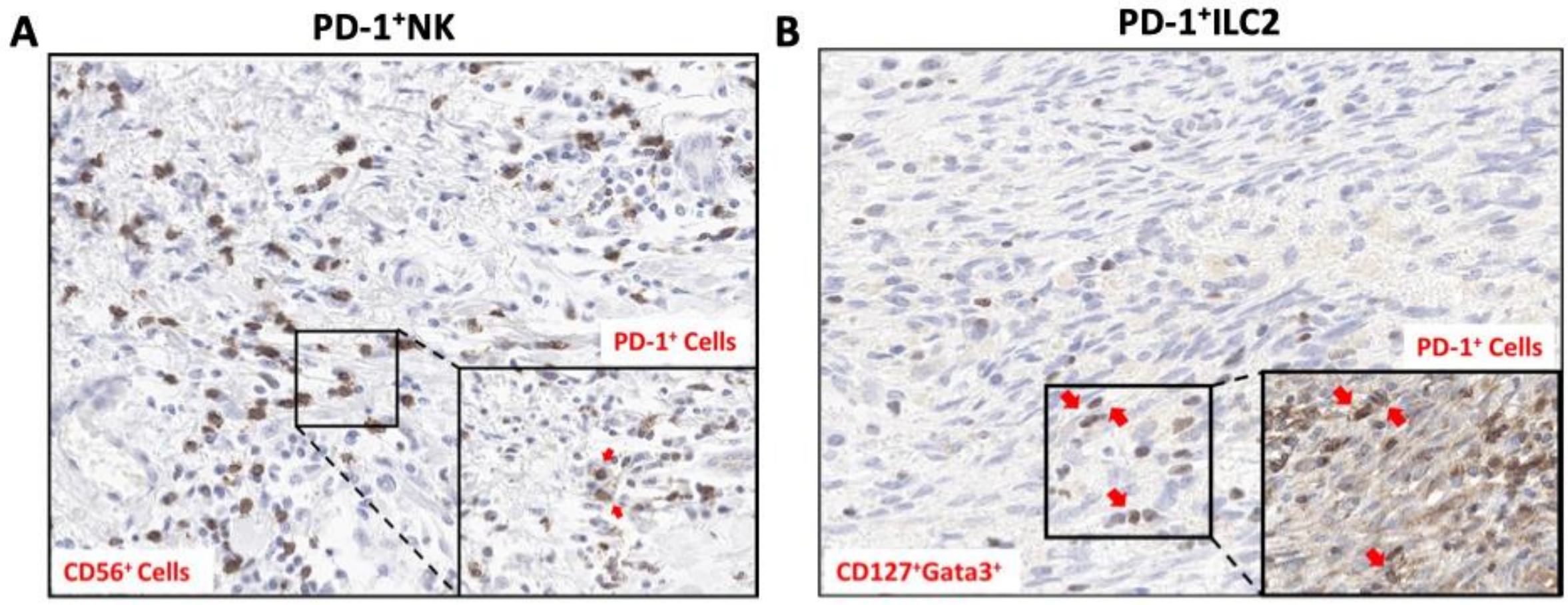

Supplementary Figure S4. Immunohistochemical detection of infiltrating $PD\text{-}1^+$NK and $PD\text{-}1^+$ILC2 cells in primary HGSOC tumors. (A and B). Representative immunohistochemistry staining of primary HGSOC tissue sections demonstrating the infiltration of $PD\text{-}1^+$NK ($CD56^+PD\text{-}1^+$) (**A**) and $PD\text{-}1^+$ILC2 ($CD127^+GATA3^+PD\text{-}1^+$) (**B**) cells. CD56 was used as the NK cell marker. ILC2s were identified by detecting $CD127^+$ cells and confirming GATA3 expression in the corresponding serial section. PD-1 expression was then assessed in matching cells, confirming the presence of $PD\text{-}1^+$ ILC2s. Insets show magnified views of the boxed areas, and arrows indicate representative $PD\text{-}1^+$ cells. We used 100 and 50 µm for magnification.
